# Chemically Defined Xeno- and Serum-Free Cell Culture Medium to Grow Human Adipose Stem Cells

**DOI:** 10.3390/cells10020466

**Published:** 2021-02-22

**Authors:** Stefano Panella, Francesco Muoio, Valentin Jossen, Yves Harder, Regine Eibl-Schindler, Tiziano Tallone

**Affiliations:** 1 Foundation for Cardiological Research and Education (FCRE), Cardiocentro Ticino Foundation, 6807 Taverne, Switzerland; stefano.panella@cardiocentro.org (S.P.); francesco.muoio@cardiocentro.org (F.M.); 2 Institute of Chemistry & Biotechnology, Competence Center of Biochemical Engineering & Cell Cultivation Technique Zurich University of Applied Sciences, 8820 Wädenswil, Switzerland; jose@zhaw.ch (V.J.); eibs@zhaw.ch (R.E.-S.); 3 Department of Plastic, Reconstructive and Aesthetic Surgery, EOC, 6900 Lugano, Switzerland; yves.harder@eoc.ch; 4 Faculty of Biomedical Sciences, Università della Svizzera Italiana, 6900 Lugano, Switzerland

**Keywords:** *UrSuppe*, defined cell culture, xeno- and serum-free cell culture, adipogenic differentiated adipose-derived stromal cells, white and beige/brown adipocyte, platelet lysate

## Abstract

Adipose tissue is an abundant source of stem cells. However, liposuction cannot yield cell quantities sufficient for direct applications in regenerative medicine. Therefore, the development of GMP-compliant ex vivo expansion protocols is required to ensure the production of a “cell drug” that is safe, reproducible, and cost-effective. Thus, we developed our own basal defined xeno- and serum-free cell culture medium (*UrSuppe*), specifically formulated to grow human adipose stem cells (hASCs). With this medium, we can directly culture the stromal vascular fraction (SVF) cells in defined cell culture conditions to obtain hASCs. Cells proliferate while remaining undifferentiated, as shown by Flow Cytometry (FACS), Quantitative Reverse Transcription PCR (RT-qPCR) assays, and their secretion products. Using the *UrSuppe* cell culture medium, maximum cell densities between 0.51 and 0.80 × 10^5^ cells/cm^2^ (=2.55–4.00 × 10^5^ cells/mL) were obtained. As the expansion of hASCs represents only the first step in a cell therapeutic protocol or further basic research studies, we formulated two chemically defined media to differentiate the expanded hASCs in white or beige/brown adipocytes. These new media could help translate research projects into the clinical application of hASCs and study ex vivo the biology in healthy and dysfunctional states of adipocytes and their precursors. Following the cell culture system developers’ practice and obvious reasons related to the formulas’ patentability, the defined media’s composition will not be disclosed in this study.

## 1. Introduction

Adipose tissue is usually classified by morphology into white, brown, or beige subsets [1,2]. The primary white adipose tissue (WAT) depot is further subdivided as either subcutaneous (SAT) or visceral (VAT). On the other hand, the minor white adipose depots include the dermal WAT (dWAT) and bone marrow adipose tissue (MAT), which are considered distinct from SAT and VAT [3]. Rodents are commonly used to study human metabolism and obesity. However, it is unclear to what extent rodent fat pads are a suitable human adipogenesis model in health and disease. Indeed, there are essential anatomical differences that should be considered [4,5]. One distinction between human and rodent VAT is that humans have a clear detectable omental WAT (oWAT), whereas rodents have a large perigonadal WAT (pgWAT). Additionally, mouse SAT is separated from dermal adipose tissue by a smooth muscle layer, whereas in humans, the SAT is continuously found with dermal adipose tissue. Finally, rodent adipose tissue deposition is strikingly dissimilar from humans, as is the fat pad composition and location associated with sex hormones [4]. A striking difference between the two species also exists regarding brown adipose tissue (BAT) regulation mechanisms or WAT “browning” [6,7]. Besides the macroscopic anatomical or morphological discrepancies, human and mouse WAT’s cellular compositions also show essential dissimilarities. One of the most striking examples of this is the marked differences in steady-state immune cell composition between human and mouse WAT [8]. This observation suggests that the two species’ adipose tissue’s critical immunoregulatory processes might be considerably different. Moreover, numerous examples at the molecular level highlight the differences between human and murine adipose tissue. For example, one of them concerns the mechanism through which glucocorticoids (GCs) affect adipogenesis. Indeed, GCs increase LMO3 (a proadipogenic factor) in human adipose stem cells (ASCs), but not in mouse primary ASCs [9]. Furthermore, in vitro cortisol treatment increases UCP1 expression in human brown adipocytes while suppressing it in mouse brown adipocytes [7]. Another example concerns the Wnt1-inducible signaling pathway protein-1 (WISP1); in humans, its expression increases during adipocyte differentiation and has a positive effect on adipogenesis, whereas in mice, it inhibits adipocyte differentiation [10,11]. Finally, estrogen sulfotransferase inhibits adipocyte differentiation in mice, but promotes adipogenesis in humans [12,13]. In contrast, Mesoderm-specific transcript (MEST) acts as an inhibitor of human adipogenesis, but stimulates murine adipogenesis [14]. Thus, the results obtained from rodent models or murine cell lines require validation in human cell culture models to demonstrate their relevance to human physiology. At this point, one might wonder if it is ultimately convenient to work with mouse models as the results cannot always be used to understand the complex biology of the human adipose tissue.

This line of reasoning can also be extended to other organs or tissues. Therefore, it is not surprising that the pharmaceutical industry has realized how dangerous it is to rely solely on animal models during the early stages of the drug discovery pipeline [15,16]. Indeed, preclinical studies based on transformed animal cell lines, conventional cell culture conditions with undefined serum-containing media, and animal models have usually proven unable to predict drug efficacy in human trials [17,18]. The same problems concern basic research; murine cell lines, extensively used over many years and until now, are not a good study model for finding remedies for the worldwide obesity pandemic and obesity-related diseases. It is essential to improve the in vitro studies by introducing primary human adipose tissue-derived cells with defined cell culture media, which offer a more relevant and versatile adipocyte biology model than murine cell lines cultured with undefined media [19].

With these concerns in mind, we developed our own basal defined serum- and xeno-free cell culture medium (*UrSuppe*), specifically formulated to grow primary human adipose stem cells (hASCs). Here, we show that we can put the stromal vascular fraction (SVF) cells extracted from human donors’ adipose tissue directly in culture with our chemically defined medium to obtain hASCs. Remarkably, this is also the case for cryopreserved cell samples (SVF or serum-free passaged hASCs). In both of these cases, the cells proliferate while preserving their undifferentiated status. We also determined the basic growth-dependent parameters of the hASCs grown in our defined medium as well as investigated on which substrate the cells grow best. As the expansion of hASCs represents only the first step in a cell therapeutic protocol or for further basic research studies, we developed two chemically defined media to differentiate the expanded hASCs in white or beige/brown adipocytes. These new chemically defined media could help translate research projects into the clinical application of hASCs or establish superior models to study the biology of human adipocytes and their precursors ex vivo. More realistically, studies with primary hASCs will contribute to an improved understanding of adipocyte biology in healthy and dysfunctional states [20,21] or their role in developing or their role in developing severe diseases, such as certain types of tumors [22,23].

## 2. Materials and Methods

The detailed description of the chemicals and reagents used in this study is shown in the online Supplementary Information Table S1.

### 2.1. Isolation and Cultivation Protocols

#### 2.1.1. Extraction of Adipo-Cutaneous Tissue

Human adipose tissue samples were obtained from tissue excess originating from surgical interventions performed in the Department of Plastic, Reconstructive and Aesthetic Surgery at the Ospedale Regionale di Lugano (Switzerland). All patients who donated their spare adipo-cutaneous tissue provided written agreement in compliance with the directives of the local Ethics Committee of the Canton of Ticino, which approved the project and its procedures (project reference number: CE 2915). Briefly, the cellular source used in this study originated from adipo-cutaneous tissue harvested from the abdominal region of female patients undergoing autologous breast reconstruction under general anesthesia. Initially, a symmetrical diamond-shaped abdominal flap depending on the deep inferior epigastric artery and its perforating vessels (DIEP-flap) was removed between the umbilicus and the pubis. After that, any excess of adipo-cutaneous tissue that was not used for breast reconstruction was packed into two sterile bags to avoid contamination and was handed over to allow for further tissue proceeding. Adipose tissue biopsies were kept at room temperature and processed within 24 h [24] to obtain the stromal vascular fraction (SVF).

#### 2.1.2. Isolation of Stromal Vascular Fraction (SVF)

Extraction of the SVF from human adipose tissue, in vitro expansion of ASCs, and their cryopreservation were in line with the ethical principles outlined in the Declaration of Helsinki and in compliance with the directives of the Ethics Committee of the Canton of Ticino (Switzerland). Briefly, adipose tissue was washed with DPBS with Ca^2+^ and Mg^2+^ (Bioconcept, Allschwil, Switzerland) in a Gosselin sterile container (Corning, New York, USA), cut into smaller pieces, and homogenized with an immersion blender. The fat was dissociated by digestion with collagenase type B, animal origin free (Worthington, Biochemical Corp., Lakewood, NJ), at a final concentration of 0.28 Wünsch U/mL for 45 min at 37 °C under constant, but gentle agitation [24]. The dissociated cells were subsequently filtered through a 100 µm and then through a 40 µm sieve cell strainer (Becton Dickinson, Franklin Lakes, NJ). All centrifugation steps were completed at 600× *g* for 10 min at room temperature. At the end of the extraction procedure, the cellular pellet was resuspended in PBS without Ca^2+^ and Mg^2+^ (Biowest, Nuaillè, Frace) with 1% injectable human albumin (CLS Behring, Bern, Switzerland) or in the cell culture medium. More complete and detailed information can be found online in the Supplementary Information (S1: Isolation of the stromal vascular fraction from human adipose tissue, Figures S1–S3).

#### 2.1.3. Cell Seeding and Culture

Cells were always and exclusively cultivated using our proprietary defined serum- and xeno-free cell culture medium, “*UrSuppe*”. The medium contains only defined molecules, some recombinant human growth factors, and injectable human albumin in the µg range. After the characterization and enumeration of the SVF cells through flow cytometry, the cells were seeded at a density of approximately 30,000 ASCs/cm^2^ in Fibronectin coated vessels (Corning, New York, USA) and kept in a humidified incubator at 37 °C and 5% CO_2_. This initial passage of the primary cell culture was referred to as passage 0 (P0). Details can be found in the Supplementary Information (S2: Characterization of the cells of the SVF from adipose tissue, S3: Initial plating of cells of the SVF for expansion, and Figure S4). Cells were maintained in culture until they achieved 75–90% confluence. They were then either collected and cryopreserved or replated in new Fibronectin coated tissue culture vessels at a density of 5000–10,000 ASCs/cm^2^. The medium was changed every 2–3 days, always keeping 20–50% of the conditioned medium, until the cells reached confluency between 75 and 90%. Cells were detached with TrypLEe Select (Life Technologies, Thermo Fisher Scientific, Waltham, USA) for 2 min at 37 °C, then washed with PBS supplemented with 1% injectable human albumin and pelleted by centrifugation at 400× *g* for 5 min. After discarding the supernatant, the cells were counted with Trypan Blue (Thermo Fisher Scientific, Waltham, USA), resuspended in *UrSuppe*, and passaged or used for various tests. Please see the Supplementary Information (S4: Basic guidelines for working with serum-free (SF) media and Figure S5) for further details and explanations.

### 2.2. Serum-Free Cryopreservation of the Cells (SVF & hASCs)

After isolation of the SVF or after detaching the expanded hASCs, the cells were resuspended in 1 mL of pre-cooled (4–8°C) freezing solution Synth-a-Freeze CTS (Thermo Fisher Scientific, Waltham, USA) at a concentration of at least 1 to 5 × 10^6^ cells/mL (or 0.5 to 2 × 10^6^ cells/mL for expanded hASCs). The cellular suspension was then transferred in a 2 mL plastic screw-capped cryotube (Nunc, Thermo Fisher Scientific, Waltham, USA) chilled on ice. Finally, the cryotubes were moved into the Mr. Frosty Cryo 1 °C Freezing Container (Nalgene, Thermo Fisher Scientific, Waltham, MA, USA) and put in a −80 °C freezer. The next day, the cryopreserved cells were transferred to a liquid nitrogen tank for final storage. In some cases, to enhance cell viability, and thus to obtain a higher yield upon thawing, the cryovials were frozen using a freezing device (Nalgene^TM^ Cryo 1 °C Freezing Container, Thermo Fisher Scientific, Waltham, MA, USA) or a controlled cooling rate protocol. The thawing protocol consists of a dilution system. The cryopreserved cell samples were quickly thawed by dilution with *UrSuppe* medium to have a final maximal DMSO (Dimethyl Sulfoxide) concentration of 1% or less. The cells were seeded without washing or centrifuging. After 5–6 h, when the cells adhered, the cell culture medium was replaced with a fresh cell culture medium. More information is reported in the Supplementary Information (S5: Cryopreservation and thawing of the SVF and Figure S6).

### 2.3. Influence of Surface Coating on hASCs Attachment and Growth

hASCs at passage P2 were seeded at a low density (~ 2′000 cells/cm^2^) in the screenMatrix 96-well plate (denovoMatrix, Dresden, Germany) and in 96-well plates without coating (TPP Techno Plastic Products AG, Trasadingen, Switzerland) as a negative control with the *UrSuppe* medium. After one week of culture, a cell proliferation assay was performed using the Cell Counting Kit 8 (Dojindo Laboraties Inc., Kumamoto, Japan) according to the manufacturer’s instruction. After the incubation with the substrate WST-8, the amount of the formazan dye generated by the activity of the cells, which is directly proportional to the number of living hASCs, was measured at 450 nm using a microplate reader (Anthos Reader 2001, Anthos Mikrosysteme GmbH, Krefeld, Germany). The average absorbance of the control wells (blank) was subtracted from that of the test wells with cells. All the data were then expressed as relative absorbance using the negative control cells as a reference value. The clean values obtained in this way were depicted in scatter plots showing the means and the standard deviations.

### 2.4. hASC Growth Characterization under Planar, Static Conditions

Two-dimensional (2D) growth characterization of previously isolated hASCs (see Table 1) was performed as duplicates (n = 2 per donor) in pre-coated T_25_-flasks (10 μg/cm^2^ r-fibronectin; Sigma Aldrich, St. Louis, MO, USA) with *UrSuppe* cell culture medium (5 mL). For this purpose, the cryopreserved, patient-derived hASCs (P0) were thawed and pre-cultured in pre-coated T_75_-flasks (10,000 hASCs/cm^2^, 37 °C, 5% CO_2_, 80% rH) in order to achieve the required cell numbers to inoculate 44 × T_25_-flasks per donor (10,000 hASCs/cm^2^, see Table 1). The hASC growth characteristics were assessed over ten days by harvesting two T_25_-flasks per donor and experiment (2 mL TrypLE Select at 37 °C, 2 min) every day. The cell density, substrate, and metabolite measurements (Glc, Lac, Amn) were carried out using a NucleoCounter NC-200 (Chemometec, Allerod, Denmark) and a Cedex Bio (Roche Diagnostics, Rotkreuz, Switzerland), respectively. Cell growth and substrate/metabolite concentrations were modeled using an unstructured growth model recently published by our groups [25,26]. Microscopic pictures (EVOS M7000, Thermo Fisher Scientific, Waltham, MA, USA) were taken daily to evaluate the cell morphology and the cell confluency. In each case, partial medium exchanges of 40% and 60% were performed for each donor on days 4 and 8. To measure the metabolite concentration after the medium exchange, 1 mL of supernatant from one T-flask was removed and substituted with 1 mL of *UrSuppe* cell culture medium. This additionally treated T-flask was used for the next day’s sampling. Detailed formulas used to calculate growth parameters can be found in Supplementary Materials Section S6.

### 2.5. Characterization of Expanded Human ASCs

#### 2.5.1. Flow Cytometry Analysis

A small aliquot of freshly extracted cells of the SVF was first counted and then diluted to a concentration of ~7.5 × 10^6^ cells/mL in *FACS buffer*: PBS supplemented with 1% injectable human albumin and 50 ng/µL of human immunoglobulins (CLS Behring, Bern, Switzerland) 40 µL (~300,000 cells) were stained with CD34-BV650 (Brilliant Violet 650), CD45-PC7, CD73-FITC (BioLegend, San Diego, CA), CD146-PE, CD36-APC (Miltenyi BioTech, Bergisch, Germany), 7-amino-actinomycin D (7-AAD) (Becton Dickinson, Franklin Lakes, NJ), and Syto 40 Blue Fluorescent Nucleic Acid Stain (Life Technologies, Thermo Fisher Scientific, Waltham, MA, USA). After 20 min of incubation, erythrocytes were lysed with 200 µL of VersaLyse solution (Beckman Coulter Inc., Pasadena, CA, USA).

Expanded and induced cells, 100 µL of the cell suspension (~50,000 cells) was pipetted into a well, and gently mixed with the appropriated antibodies combination. Each test contained a different mixture of the following antibodies: CD26-FITC, CD73-FITC, CD90-APC, CD95-FITC, CD105-PE, CD142-APC (BioLegend, San Diego, CA, USA), CD34-FITC, CD36-FITC, CD137-APC, CD146-PE (Miltenyi BioTech, Bergisch, Germany), CD55-BV421, CD248-BV421, 7-AAD (Becton Dickinson, Franklin Lakes, NJ, USA), CD54-PE, CD140a-APC, and CD106-PC7 (Thermo Fisher Scientific, Waltham, MA, USA). Cells were incubated for 20 min at room temperature in the dark. Finally, the sample was diluted with 100 µL FACS buffer before the acquisition of the data.

All antibodies were titrated to optimize the signal-to-noise ratio and used at a concentration of 50 ng/test. Isotype controls and specific mAbs were used at the same final concentrations. Further information about the antibodies used for this analysis can be found in the Supplementary Information Table S2. Stained cells were analyzed using the Cytoflex flow cytometer (Beckman Coulter Inc., Pasadena, CA, USA) and, subsequently, the raw data were processed with the Kaluza software (Beckman Coulter Inc., Pasadena, CA, USA). Fluorescence compensation was assessed using single stained control particles (VersaComp Antibody Capture Bead Kit, Beckman Coulter Inc., Pasadena, CA, USA) or cells to determine the labeled antibodies’ spill-over coefficients for the stainings.

#### 2.5.2. Quantitative Reverse Transcription PCR (RT-qPCR) Analysis

Total RNAs were purified using the Nucleospin^®^RNA kit (Macherey-Nagel, Düren, Germany) according to the manufacturer’s instructions. The kit’s purification procedure also includes an on-column digestion step with DNase I. RNA purity and quantity were assessed with the NanoDrop device (Thermo Fisher Scientific, Waltham, USA) and total RNA integrity was periodically verified by conventional agarose gel electrophoresis. cDNA was obtained from 400 ng RNA using the GoScriptTM Reverse Transcription System (Promega, Madison, WI). The protocol can be found in the Supplementary Information (Table S3). RT-qPCR of the following genes *PREF1, SOX9*, *ZFP521*, *WISP2*, *NOTCH1*, *DLL1*, *PPARG*, *ZFP423*, *RUNX2*, *CD146*, *WISP1*, *DKK1*, *CD34*, *CD36*, *α-KLOTHO*, *β-KLOTHO, CD26*, *CD55*, *CD142*, *CD248*, *FABP4*, *ADIPONECTIN*, *LEPTIN*, *UCP1,* and *PGC1α* was performed using the SsoAdvancedTMUniversal SYBR^®^Green Supermix kit (Biorad, Hercules, CA) and detected with the CFX Connect system (Biorad, Hercules, CA, USA). RT-qPCR was performed using 20 ng of cDNA for each gene, and *ACTB* was used as an internal control. Each primer pair product was checked for proper amplification by agarose gel electrophoresis. We exclusively used primers, which gave rise to single sharp bands of the expected size. Primer sequences, temperature, and cycle conditions can be found in Supplementary Information (Tables S4 and S5). Data were analyzed with the CFX software to calculate the relative fold gene expression using the formula 2^−ΔΔCt^.

### 2.6. hASCs’ Differentiation

#### 2.6.1. Adipogenic Differentiation—WAT and BAT

Two completely chemically defined serum- and xeno-free differentiation media were developed, *UrSuppe*-WAT: to induce hASCs in white adipose tissue (US-WAT); and *UrSuppe*-BAT: to induce hASCs in beige/brown adipose tissue (US-BAT). Both contain specific recombinant human growth factors to induce the maturation in white or beige adipocytes, dexamethasone, and injectable albumin in the µg range. For adipogenic differentiation, hASCs were seeded at P2 with *UrSuppe* medium in collagen I coated 60 mm Petri dishes (Greiner Bio-One, Kremsmünster, Austria). After reaching full confluency, the cells were cultured in an adipogenic differentiation medium (US-WAT or US-BAT). The medium was changed every four days up to a total incubation period of ten days for WAT and seven days for BAT.

#### 2.6.2. Oil Red O Evaluation

Oil Red O (Sigma-Aldrich Inc., St. Louis, MO) staining was performed to examine cytoplasmic lipid vesicles during adipogenesis. Briefly, the Oil Red O stock solution (3% [*w*/*v*] in isopropanol, Sigma-Aldrich Inc., St. Louis, MO) was diluted 3:2 with deionized water, and filtrated to prepare the Oil Red O working solution. The samples were washed with PBS, fixed with a fixing solution (2% formaldehyde, 0.2% glutaraldehyde in PBS, Sigma-Aldrich Inc., St. Louis, MO, USA) for 15 min, washed with deionized water, and stained for 5 min with Oil Red O working solution. Residual dye was washed out with deionized water and samples were directly observed by light microscopy. Cells were incubated in the dark for 15 min with a solution of 10 µg/mL Hoechst (Molecular Probes, Eugene, OR, USA) diluted in PBS. The vessels were then washed with PBS, and nuclei were counted using an automated Lionheart FX system (BioTek Instruments Inc., Winooski, VT, USA).

To evaluate the induction efficiency, plates were incubated with 100% isopropanol for 5 min, with gentle rocking, to extract the Oil Red O dye. The liquid phase was then transferred to spectrophotometer cuvettes, and the absorbance was read at 518 nm (Specord40, Analytic Jena, Germany), using 100% isopropanol as a control to subtract the background signal. Evaluation of the adipogenic differentiation is based on quantifying the Oil Red O absorbance, normalized for the number of cells present in the cell culture vessel.

#### 2.6.3. JC-10 Staining and Fluorescence Microscopy

The JC-10 assay provides a robust assay method for monitoring mitochondria membrane potential (MPP) changes. This molecule is analog to JC-1 with improved solubility in aqueous media and increased sensitivity to detect subtler changes in matrix metalloproteases (MMP). Beige and brown adipocytes are characterized by the fact that they can depolarize the mitochondrial membrane potential, leading to the dissipation of chemical energy into heat. In normal cells, JC-10 accumulates in mitochondria, selectively generating an orange J-aggregate emission profile (590 nm). However, upon maturation into a beige or brown adipocyte, as the membrane potential decreases, JC-10 monomers are generated, resulting in a shift to green emission (525 nm). Thus, this is a very convenient method of recording the switch from undifferentiated ASCs into brown/beige adipocytes. Experimentally, the test was carried out as follows. After induction of the hASCs with US-WAT or US-BAT medium, the cells were washed twice with PBS and then incubated in a basal medium containing JC-10 (Adipogen, San Diego, CA, USA. Stock solution 1000× in DMSO: 10 mM), and diluted according to the manufacturer’s instructions (Final concentration: 10 µM), for 30 min at 37 °C. After this step, the cells were washed with PBS to remove an excess of reagent. Then, samples were directly observed under an inverted fluorescence microscope (UV laser 405 nm) Nikon Eclipse Ti (Nikon, Minato, Tokyo, Japan).

#### 2.6.4. Characterization of Human Differentiated ASCs: RT-qPCR Analysis

At the end of the induction or culture period, the medium was removed, and the cells were washed with PBS. Then, 1.5 mL of *extraction buffer* (7M Urea, 1% SDS, 0.35 M NaCl, all chemicals from Sigma-Aldrich Inc., St. Louis, MO) was added to the cell culture vessel, and the lysate was collected with the help of a cell spatula (TPP, Trasadingen, Switzerland). As quickly as possible, the lysate was transferred into a 5 mL ED Eppendorf tube (Eppendorf, Hamburg, Germany) containing 1.5 mL of phenol/chloroform/isoamyl alcohol 25:24:1 solution (all chemicals from Sigma-Aldrich Inc., St. Louis, MO, USA) and mixed vigorously. The aqueous phase was then transferred to a new ED tube containing 3.5 mL of 100% EtOH (Sigma-Aldrich, St. Louis, MO, USA), mixed, and centrifuged at 2200× *g* for 10 min at room temperature (RT). The supernatant was discarded, and the pellet was first washed with 100% EtOH and then resuspended in 100 µL of Accugene water (Lonza, Basel, Switzerland). Finally, the pellet was processed as indicated in the RT-qPCR analysis section (Section 2.5.2).

#### 2.6.5. Secretome Profiler

The conditioned cell culture medium (*UrSuppe*, US-WAT, and US-BAT) was analyzed to detect secreted cell factors. Each conditioned medium was filtered through a 0.22 µm syringe mesh (Jet Biofil, Guangzhou, China) to remove cellular debris, then 1 mL of each supernatant was incubated overnight with a nitrocellulose membrane containing many different capture antibodies. The array procedure was carried out according to the instructions provided with the Proteome Profiler Human Adipokine Array Kit (R&D Systems, Minneapolis, MN, USA). The secondary biotinylated detection antibodies were highlighted by the use of Streptavidin conjugated IRDye 800 CW (Li-Cor Corporate, Lincoln, NE, USA). Positive signals were detected after scanning the membrane using an Odyssey^®^CLX Imaging System (Li-Cor Corporate, Lincoln, NE, USA). Data were analyzed using Li-Cor software to evaluate the signal intensity ratio between the different adipokine spots and the positive reference spot.

### 2.7. Statistical Analysis

Statistical analysis was performed using SPSS or Graph Pad Prism software. The differences between groups were evaluated with a one-way analysis of variance (ANOVA) and Bonferroni post-hoc test. The assumption of the analysis was checked: normal distribution and homogeneity of the variance. *p*-values lower than 0.05 were considered as statistically significant.

## 3. Results

### 3.1. Comprehensive Phenotypic Characterization of Freshly Extracted Cells of the SVF

**Figure 1:**
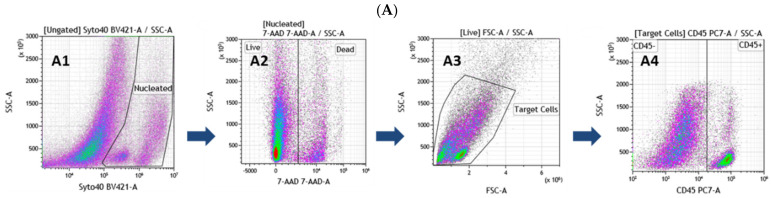

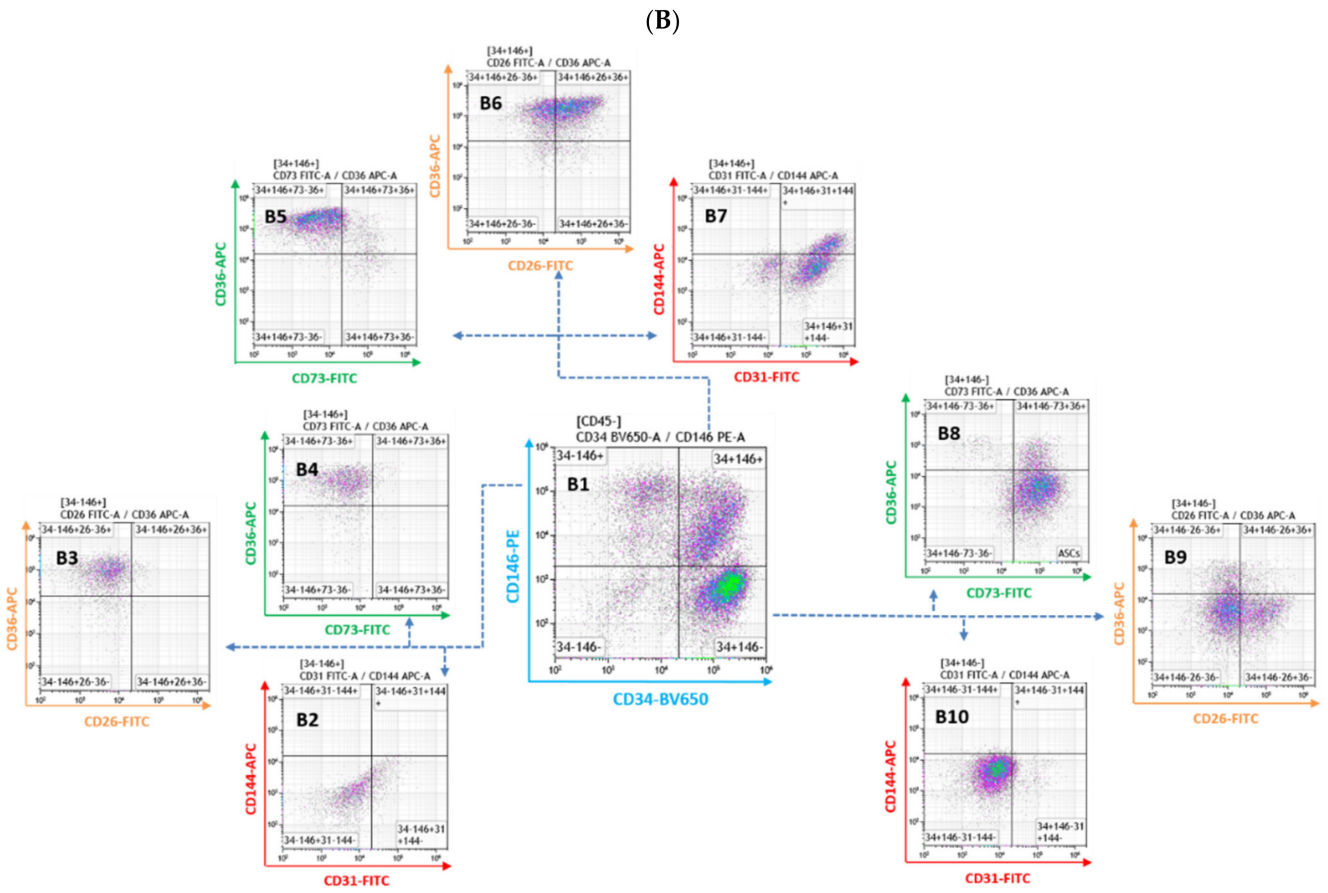
Representative flow cytometry analysis of stromal vascular fraction (SVF) cells based on our seven-color staining procedure. (**A**) The gating strategy used to find viable target cells is shown. Syto 40 marks all nucleated cells, and 7-AAD discriminates between living and dead (plot **A1** and **A2**, respectively). In the next two gates (plot **A3** and **A4**, respectively), we selected cells that are relatively small and CD45^−^. (**B**) Representative analysis of the SVF cells extracted from human subcutaneous adipose tissue. CD45^−^ target cells were first analyzed for the expression of CD34-BV650 and CD146-PE. This leads to four sub-populations of cells (plot **B1**). Except for the lower left quadrant of plot B1, the cells present in the three other quadrants were further investigated for the expression of three pairs of new markers: (1) CD31-FITC/CD144-APC (plots **B2**, **B7**, & **B10**). (2) CD26-FITC/CD36-APC (plots **B3**, **B6**, **B9**). (3) CD73-FITC/CD36-APC (plots **B4**, **B5**, **B8**). Data in A and B are from a representative patient. See the Supplementary Information (S2: Characterization of the cells of the SVF from adipose tissue and Table S2) for more details about the markers used for this flow cytometry analysis.

To culture SVF cells immediately after extraction in the defined serum-free conditions, it is crucial to know the type and the relative number of cells present in the preparation to seed them at the optimal density. If the appropriate density is not hit, it is unlikely to obtain a confluent hASC P0 culture. Therefore, based on the existing literature [27,28,29], we designed a new combination of cell surface markers and intracellular stains to characterized freshly extracted SVF cells. This analysis is made more difficult by the presence of a large amount of debris in a cell suspension. As shown in Figure 1A, the use of Syto 40 Blue Fluorescent Nucleic Acid Stain (stains all nucleated cells), of 7-AAD (live/dead discrimination), and the gating for small size cells, helped us to define the nucleated living target cells. Subsequently, we use an anti-CD45-PC7 antibody to differentiate stromal from blood cells (Figure 1A4). Finally, the CD45 negative stromal cells were analyzed for the expression of CD34 and CD146, leading to the biparametric plot B1 shown in Figure 1B. This allowed for the determination of four different populations: (1) CD34^+^, CD45^−^, CD146^−^: *ASCs*; (2) CD34^+^, CD45^−^, CD146^+^: Endothelial cells; (3) CD34^−^, CD45^−^, CD146^+^: Mural cells; (4) CD34^−^, CD45^−^, CD146^−^: Other not defined cells. With the five-color flow cytometry assay, we were able to count the hASCs present in the sample. We could use this information to seed the cells at an optimal density, making it almost certain that the hASCs would take root in the defined serum-free conditions.

CD34 and CD146 are highly informative markers because they are expressed starting from early progenitors to more differentiated and mature adipocytes [30,31,32,33,34,35]. However, unfortunately, they are also present on the surface of several different types of adult vascular wall resident progenitor or mature cells [36,37,38], giving rise to diverse populations of cells with varying degrees of stemness or maturation. Therefore, it is judicious to add two additional colors to the basal panel to deepen the analysis and better characterize the cells of the SVF. Thus, the results obtained with three tests using three different seven-colors panels are shown in Figure 1B. Each subpopulation of plot B1, obtained with the first five colors, can be further analyzed with the addition of two additional antibodies. The rationale for choosing these three combinations is as follows:

- CD26-FITC/CD36-APC, “cell fate” panel. Recently, the CD26 marker was found on the surface of early adipocyte progenitor cells. In contrast, the CD36 surface marker is directly involved in adipocyte differentiation, and its expression level increases gradually when ASCs are induced to mature into adipocytes [30,33,39,40,41]. Therefore, these two markers allow for the discrimination of early progenitors from more differentiated cells.
- CD73-FITC/CD36-APC, “ASCs characterization” panel. CD73 belongs to the canonical “*MSCs*” markers, and recently, it has been demonstrated that it can mark multipotent stromal cells [42]. This markers combination is beneficial for identifying early progenitors, and in combination with CD36, it is possible to exclude more differentiated and mature cells and to obtain a more precise count of the ASCs present in the SVF.
- CD31-FITC/CD144-APC, “endothelial cells” panel. CD31(PECAM-1) is a classical pan-endothelial cells marker expressed constitutively on early and mature vascular endothelial cells [43]. On the other hand, CD144 (VE-Cadherin) is a strictly endothelial-specific adhesion molecule located at the junction between endothelial cells. It is indispensable to the correct organization of the vasculature and for endothelial assembly [44]. As a result, these two antibodies allow for the identification of cells with an endothelial origin.

Thus, the further analysis of the four sub-populations defined in plot B1 (Figure 1B) with the three combinations of antibodies described above leads to these observations:

- The CD34^+^, CD45^−^, and CD146^−^ cells are negative for endothelial markers (plot B10) and the majority of them are positive for CD73 and negative for CD36 (plot B8). Finally, a significant number of these cells are CD26^+^/CD36^−^ (plot B9). This profile is compatible with early progenitor cells, and for this reason, we named the population of the lower right quadrant of the plot B1 as ASCs.
- The majority of the CD34^+^, CD45^−^, and CD146^+^ cells are positive for the endothelial markers (plot B7). Some of them are also CD144^+^, and thus mature endothelial cells. They also strongly express CD36 and are CD73 negative (plot B5). Interestingly, blot B6 shows that the majority of these cells are CD26^+^/CD36^+^. CD26 has been recently described as a marker for early precursors [45]. Seeing it co-expressed with CD36 [40], a typical marker present on mature or differentiating cells, is surprising and worth highlighting. Taken together, the upper right quadrant of plot B1 mainly contains cells of endothelial origin at different stages of maturation.
- The majority of the CD34^−^, CD45^−^, and CD146^+^ cells are negative for the tested markers, except for CD36 (plot B2, B3, and B4), which is highly expressed. We, therefore, labeled the cells of the upper left quadrant of plot B1 as mural cells.
- The cells of the lower left quadrant (CD34^−^, CD45^−^, and CD146^−^) of plot B1 are not defined and have not been further analyzed.

In conclusion, we have developed an effective protocol for the flow cytometric analysis of cells extracted from the human subcutaneous tissue, which allows us to divide the SVF cells into well-defined sub-populations. Given the growing heterogeneity discovered between and within the ASC populations, a clear definition of the cell type present in the sample will become increasingly important. This knowledge is also fundamental for technical reasons. Indeed, before putting freshly extracted SVF cells in culture under defined xeno- and serum-free conditions, it is essential to identify and enumerate the putative hASCs present in the preparation. This information is necessary to seed the SVF cells at an optimal density, allowing their cultivation from the beginning under defined conditions without serum or other not defined additives such as the platelet lysate. This is the most appropriate strategy if one wants to be (almost) sure of obtaining a confluent culture of hASCs at passage P0.

### 3.2. Influence of Surface Coating on hASCs’ Attachment and Growth

**Figure 2:**
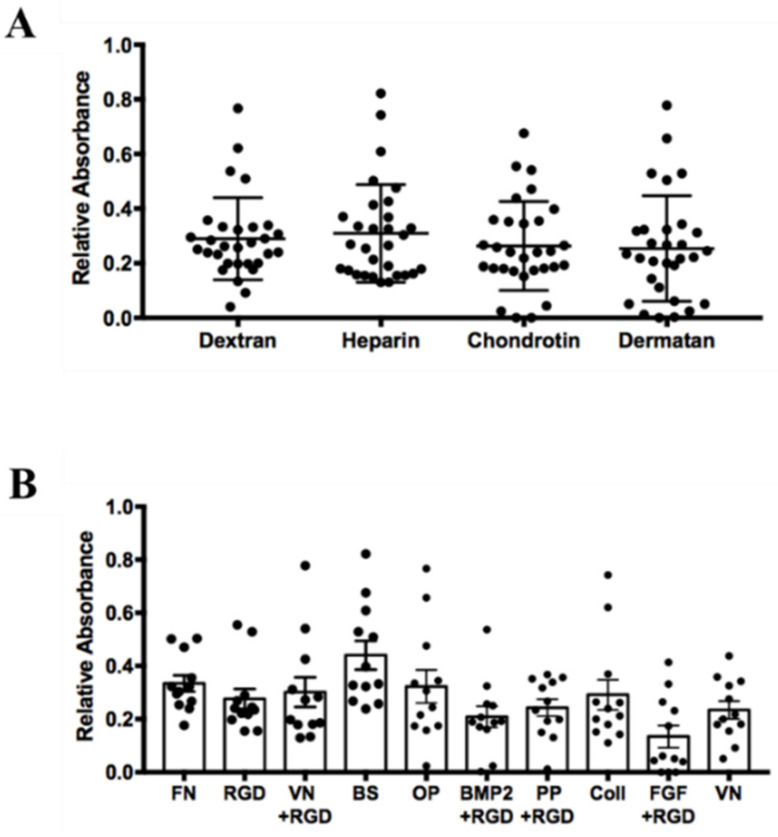
Identifying attachment substrates necessary for human adipose stem cells’ (hASCs) adherence to cell culture surface, spreading, and growth under serum-free conditions. Human ASCs obtained from three different donors were used to screen a 96-well plate coated with 24 different peptides non-covalently linked to four different highly sulphated glycosaminoglycans (GAGs: synthetic dextran sulfate, heparin, chondroitin, and dermatan) [47,48]. The cells’ growth in the 96-well plate was evaluated using a commercial colorimetric cell viability assay. (**A**) Values of relative absorbance measured at 450 nm and classified according to the four types of sulphated GAGs (synthetic dextran sulfate, heparin, chondroitin, and dermatan) were used as a backbone for the formation of the different coating matrices. The four GAGs do not have a great influence on cell attachment and growth. (**B**) Ten different peptides non-covalently linked to highly sulphated GAGs strongly influence the ability of hASCs to attach and grow in the test micro-wells. The results obtained with the 14 poor-performing peptide-combinations are not shown. As in Figure 2A, cell proliferation was measured using a commercial colorimetric kit. Histograms show the means ± standard deviations of the measured relative absorbances for hASCs obtained from three different donors. FN = fibronectin; VN = vitronectin; BS = bone sialoprotein; OP = osteopontin peptide; BMP2 = bone morphogenetic protein; PP = perlacan peptide; ColI = collagen I peptide; FGF = fibroblast growth factor.

Fetal Bovine Serum (FBS) contains attachment and spreading factors. Therefore, it is often not necessary to coat the culture vessels when working with traditional serum-containing media. On the other hand, working in serum-free conditions requires tissue culture vessels pre-coated with extracellular matrix (ECM) components. As the interaction of the cells with the ECM can affect their growth [46], we wanted to determine which substrate performs best with hASCs cultured in our serum-free cell culture medium. For this purpose, we used the “screenMatrix” (denovoMatrix GmbH, Dresden, Germany), which consists of a 96-well plate coated with 24 different biologically relevant peptides non-covalently linked to four different highly sulphated glycosaminoglycans (GAGs): synthetic dextran sulfate, heparin, chondroitin, and dermatan [47,48]. hASCs obtained from three different donors were tested independently and the results are summarized in Figure 2. The cells’ growth in the 96 wells was monitored using a commercial colorimetric cell viability assay [49] as described in the Materials and Methods. We first grouped all the absorbance values measured at 450 nm according to the four types of sulphated GAGs used to form the different coating matrices (see Figure 2A). As a result, the four GAGs used as an anchoring matrix for the 24 types of peptides do not significantly influence the capacity to attach and promote the growth of the ASCs in serum-free conditions. On the other hand, the results revealed the importance of the peptides to culture these cells successfully. Indeed, among the 24 types of tested peptides, 14 ultimately failed to sustain the attachment and the growth of hASCs. Low performing peptides belonged to the laminin family (laminin peptide 1–7, and laminin peptide 7 + RGD), osteocalcin peptide, E-cadherin peptide, tenascin peptide 1 and 2, TGF peptide, NCAM peptide, and collagen IV peptide (data not shown). Figure 2B shows the results of the 10 peptides that allow for good adhesion and growth of the cells. Although the best result was achieved with the bone sialoprotein (BS) peptide followed by the fibronectin (FN) peptide, we decide to use the latter because several FN coated cell culture vessels are already commercially available. Furthermore, it has been reported that FN, in combination with Pref-1, inhibits adipocyte differentiation [50,51]. Therefore, FN or FN-derived peptides are very appropriate substrates for growing and expanding hASCs while maintaining a high degree of stemness. For this reason, we routinely culture hASCs on Corning PureCoat™ ECM Mimetic Fibronectin Peptide plasticware [52], and all experiments of this study were performed using cells grown and expanded in serum-free conditions on this type of vessels.

### 3.3. hASC Growth under Planar, Static Conditions

**Figure 3:**
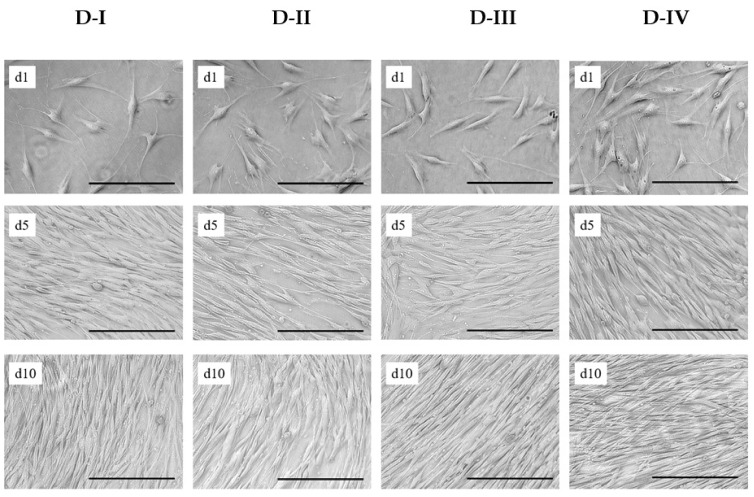
Light microscopic pictures of patient-derived hASCs during cell growth in T25-flasks. Scale bar = 275 μm. d1 = after cell attachment, d5 = nearly fully confluent, d10 = hyperconfluent.

Figure 3 shows light microscopic images of patient-derived hASCs during the growth characterization study in the T_25_-flasks. It is recognizable that the hASCs attached well to the fibronectin-coated surface under serum-free conditions. After 4–6 h, nearly all inoculated cells (viability ≥ 96%) spread out and were mitogenically active. The cells exhibited a fibroblast-like or fibroblastoid cell morphology, with minimum and maximum cell diameters in the range of 10 μm to 69 μm and 25 μm to 315 μm (on day 1). Interestingly, the average cell sizes of the hASCs from the different donors were slightly different. Cell size analysis of detached cells (hASCs in suspension) showed that hASCs from D-I (18.1 ± 0.2 μm) and D-III (18.2 ± 0.2 μm) were on average 10% smaller than those from D-II (19.5 ± 0.3 μm) and D-IV (19.4 ± 0.1 μm). In all cases, cell confluency increased immediately after the cell attachment phase as a cell number function. Cell confluency of nearly 80% to 90% was achieved by day 5. After day 5, the increase in cell confluency slowed down owing to the reduced cell proliferation rate caused by the higher incidence of cell contact inhibition. Hyperconfluent conditions were achieved in all cases by the end of the cell growth characterization study (day 10). From a visual perspective, no significant differences in morphology (i.e., shape, granularity) were found between the four donors during the culture time under the serum-free conditions.

**Figure 4:**
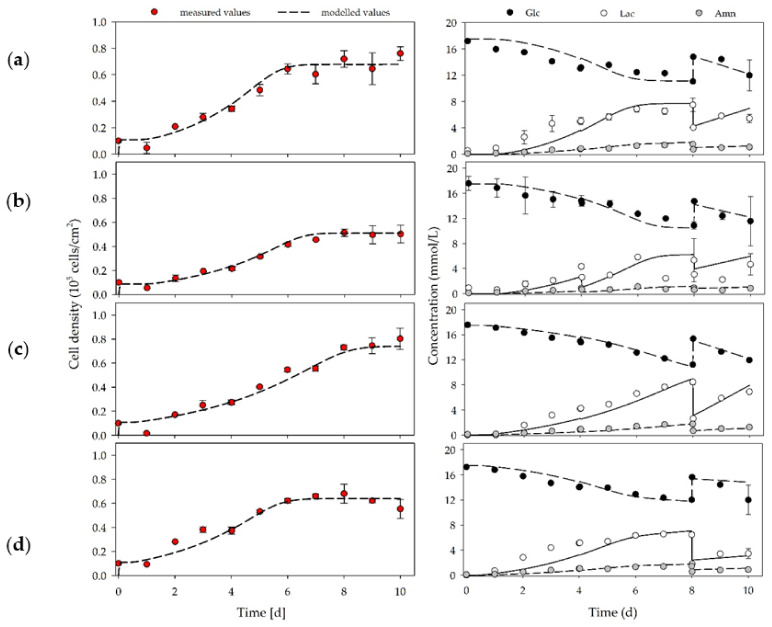
Time-dependent profiles of cell densities (left) and substrate/metabolite concentrations (right) in T_25_-flasks (n = 2 per donor). D-I (**a**), D-II (**b**), D-III (**c**), and D-IV (**d**). Partial medium exchanges of 40% and 60% were performed on days 4 and 8, respectively. The symbols represent the experimentally measured values collected from offline measurements. The lines represent the simulated time courses.

Figure 4a–d shows the cell density’s time-dependent profiles and the substrate and metabolite concentration. It is clear that, in all cases, the hASCs followed a classical exponential growth curve. The highest cell densities were achieved with 0.76 ± 0.05 × 10^5^ hASCs/cm^2^ (=3.80 ± 0.25 × 10^5^ hASCs/mL) and 0.80 ± 0.09 × 10^5^ hASCs/cm^2^ (=4.00 ± 0.45 × 10^5^ hASCs/mL) for donor D-I and D-III, respectively. The maximum cell densities of D-II and D-IV were at 0.51 ± 0.03 × 10^5^ hASCs/cm^2^ (=2.55 ± 0.15 × 10^5^ hASCs/mL) and 0.68 ± 0.08 × 10^5^ hASCs/cm^2^ (=3.40 ± 0.40 × 10^5^ hASCs/mL), on average 35% and 13% lower, respectively. During the culture period, cell viability was, in all cases, always >90%. Owing to the cells’ metabolic activity during the growth phase, glucose was consumed, and lactate and ammonia were produced. In all cases, the glucose concentration did not drop below 11 mmol/L owing to the medium’s regular partial exchanges. The maximum lactate and ammonia concentrations were measured to range from 5.83 to 8.44 mmol/L and 1.13 to 1.76 mmol/L, respectively, in all cultures. According to Higuera et al., 2009 [53]; He et al., 2013 [54]; Chen et al., 2009 [55]; and D. Schop et al., 2009 [56] and 2010 [57], lactate and ammonia did not, however, reach growth-inhibiting concentrations (*Lac* = 25–35 mM, *Amn* = 2.5 mM). The unstructured growth model well approximated time courses of the cell densities, the substrate, and metabolite concentrations. Thus, the results highlight the exponential growth characteristics and the Monod-type growth behavior of the hASCs under the serum-free conditions in the T_25_-flask cultures.

Table 2 summarizes the calculated growth-dependent parameters for the different donors (D-I to D-IV). The achieved maximum cell densities corresponded to the maximum population doubling level (PDL) and expansion factor (EF) in the range of 2.4 to 3.0 and 5.1 to 8.0, respectively. Interestingly, the specific growth rate was highest for D-I (0.45 ± 0.01 d^−1^) and D-IV (0.38 ± 0.02 d^−1^). Thus, high growth rates do not necessarily result in the highest cell densities because of the limited growth area available. Specific growth rates for D-II and D-III were 0.36 ± 0.02 d^−1^ and 0.30 ± 0.03 d^−1^, respectively. Salzig et al., 2016 [58] reported specific growth rates for human bone marrow-derived mesenchymal stem cells (hBM-MSCs) cultivated in a serum-free culture medium in the range of 0.38 to 0.45 d^−1^ (*t_d_* = 36.9 to 43.7 h). Comparable specific growth rates of 0.31 to 0.47 d^−1^ (*t_d_* = 35.4 to 53.6 h) were also reported by Heathman et al., 2015 [59] and Jossen et al., 2020 [25]. Specific glucose consumption rates were between 0.30 and 0.74 pmol/cell/d for the investigated donors and, therefore, in a close range to values reported by Jossen et al., 2020. Owing to the metabolization of glucose, specific lactate production rates of between 0.43 and 1.19 pmol/cell/d were calculated and resulted in Y_Lac/Glc_ of between 1.22 and 1.60. According to our expectations, glucose was mainly metabolized into lactate via the glycolysis pathway. Hence, only little energy was gained by the cells from OXPHOS, which was in agreement with the literature data (Sart et al., 2014 [60]). The specific ammonia production was in the range between 0.09 and 0.26 pmol/cell/d.

### 3.4. Characterization of Human Expanded ASCs

#### 3.4.1. Flow Cytometry Analysis of hASCs Grown in *UrSuppe* Medium

**Figure 5:**
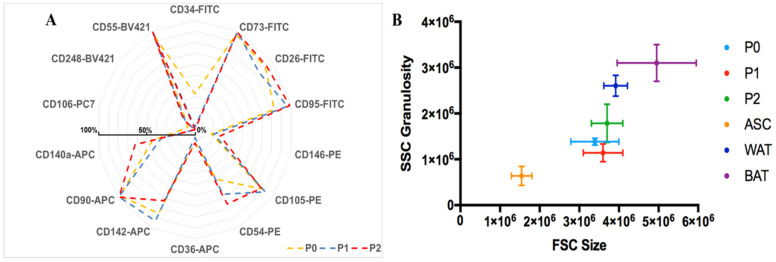
Flow cytometry analysis of cells at P0, P1, and P2 (**A**) Flow cytometry expression profile of selected markers. Data represent an average of three different samples. During culture passaging, the profile of the cells remains similar except for CD34. Data of positive cells are calculated on specific isotype controls. (**B**) Size distribution after isolation, during passages, and after differentiation (FCS: forward scatter and SSC: side scatter). P0, P1, P2: Indication of the passage number of hASCs grown in *UrSuppe* medium; ASCs found in the SVF as described and explained in Section 3.1; white adipose tissue (WAT) and beige adipose tissue (BAT) represent white or beige induction, respectively. Each point’s coordinate is the mean of three different samples; bars represent standard deviation.

The growth rate is not the only parameter that is important in the cell culture process. In fact, a crucial feature for a stem cell culture medium is its ability to “seal the undifferentiated status” and prevent slow and spontaneous differentiation of the cells during the in vitro culture and amplification phase. Thus, using flow cytometry during passage P0, P1, and P2, we followed some surface markers’ expression, which may indicate whether the hASCs display a stable phenotype or are drifting spontaneously away and starting to differentiate into early adipocyte. Unfortunately, the canonical markers [27] used for characterization of “mesenchymal stem cells” (CD73, CD90, CD105) are not sufficient to clearly distinguish hASCs from other cells [61] and to evaluate their degree of stemness. Therefore, we decided to include some recently described markers in our flow cytometry panel [45,62,63], which are expressed by early precursors and can help to sketch lineage hierarchy relationships between the cells. As shown in Figure 5A, the canonical markers CD73, CD90, CD105, the new ones, as well as CD26, CD54, CD55, CD140a, and CD142, are highly expressed. Most importantly, all these markers do not change very much during the passaging of the cells. The CD34 marker, which typically characterizes freshly extracted hASCs, tends to disappear during passaging [32,64]. Given that this marker, together with CD146, is associated with adipogenic differentiation [30,33], its fast decrease during passaging can be regarded positively and suggests that the in vitro conditions promote an early precursors state of the cultured cells. hASCs can be considered negative for CD106, and its expression does not increase during culture. This marker is used to discriminate hASCs from BM-MSCs, which, on the other hand, are associated with CD106^+^ [27]. CD36 is a very useful surface antigen because it labels progenitor cells with a particular susceptibility to undergo terminal adipogenic differentiation. Its expression is correlated with an increase in intracellular neutral lipid content [40,41]. Furthermore, it is also the ideal marker to determine if cells spontaneously start to differentiate during the culture time because it is highly expressed in mature adipocytes [30,39]. As shown in Figure 5A, this is not the case, and CD36 expression remains very low during the passaging of the hASCs. Another marker with high and stable expression is CD95 (Fas Receptor, FasR) [65]. This observation suggests that the cells can be eliminated by activating the natural apoptotic program. CD248 is involved in hypoxic regulation, angiogenesis, inflammation, and reorganization of the extracellular matrix. It is expressed at low levels in stromal and perivascular cells, but increased in the setting of tumor growth, inflammation, and injuries associated with fibrosis. Lack of CD248 in mice confers protection against the growth of some tumors and several inflammatory diseases [66]. Therefore, this is a marker that labels pathophysiological or malfunctioning cells. As shown in Figure 5A, the number of CD248 positive hASCs is very low and remains constant during the passaging of the cells. The impact of the *UrSuppe* medium on hASCs’ morphology and phenotype can also be monitored by following the shift in cell size and granularity during culturing of the cells. Using flow cytometry, the size can be measured by means of forward scatter light, and granularity by side scatter light. Normally, stem cells are small in size and have a low degree of granularity. When they start to differentiate or go into senescence, these two parameters increase [67,68]. Figure 5B displays the analysis of many measurements of hASCs obtained from several donors. Interestingly, the size of the cells slightly increases during the transition from P0 to P1, while the granularity decreases. On the other hand, during the transition from P1 to P2, both parameters increase compared with P0 or P1. Taken together, we conclude that our cell culture medium *UrSuppe* is able to support the growth of hASCs right from the beginning in defined serum-free conditions by maintaining a high expression of markers associated with an undifferentiated status and constantly keeping some markers that indicate a low progressive differentiation of hASCs.

#### 3.4.2. Expression Levels of Some Marker Genes Measured by RT-qPCR

**Figure 6:**
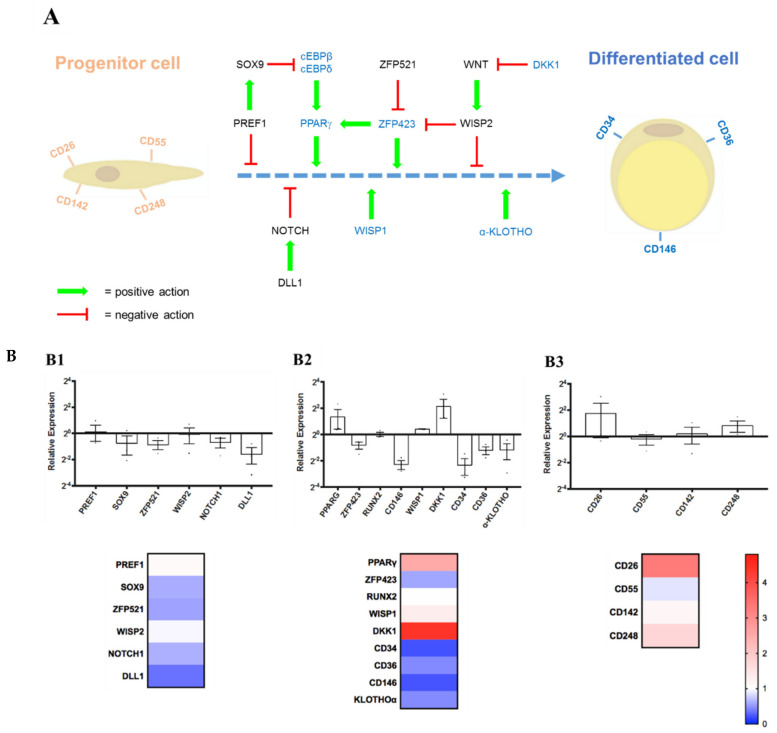
(**A**) Factors that positively or negatively regulate the adipocyte differentiation process. Different regulatory proteins act at early and later stages to control adipogenesis. (**B**) Expression levels measured by RT-qPCR of genes involved in cell stemness or cell differentiation: (**B1**) stemness maintenance genes; (**B2**) differentiation regulators/markers; and (**B3**) lineage hierarchy markers. Primary hASCs from three different donors were analyzed at passages P0, P1, and P2. The three upper bar graphs show the relative fold-expression change of the different markers at P2 compared with hASCs at P0 (n = 3, error bars represent S.E.M.). The three lower figures represent the same data as the heatmap for the three different categories.

Similar to flow cytometry, we used RT-qPCR to monitor stemness or differentiation-related genes. The cells used for this study were extracted from human subcutaneous adipose tissue. Thus, we reasoned that the default differentiation pathway of these cells should be towards adipogenesis in spontaneous unwanted and uncontrolled maturation. In recent years, several essential genes have been discovered that are important for maintaining the stemness of hASCs, while others are essential for the differentiation and maturation of these cells. A schematic representation of adipogenesis is shown in Figure 6A. Therefore, some of these genes are very useful as markers in RT-qPCR assays to verify the differentiation status of the in vitro expanded hASCs. Further information and references about the marker genes used for this study can be found in Supplementary Information (Tables S6–S8).

To facilitate analysis, the selected genes were subdivided into three groups:

(1) Stemness Maintenance Genes: *PREF1*, *SOX9*, *ZFP521*, *WISP2*, *NOTCH1*, *DLL1;*
(2) Differentiation Regulators/Markers: *PPARG*, *ZFP423*, *RUNX2*, *CD146*, *WISP1*, *DKK1*, *CD34*, *CD36*, *α-KLOTHO*;
(3) Lineage Hierarchy Markers: *CD26*, *CD55*, *CD142*, *CD248.*

Figure 6B shows the results of the RT-qPCR measurements. In the category “Stemness Maintenance” (Figure 6B1), the expression profile of these genes at passage P2 was not significantly different from that at P0. The level of *PREF1* and *WISP2* was nearly unchanged. In contrast, the others showed only slight reductions, represented in the corresponding heat map by only white color or different shades of blue. In the second category, “Differentiation Regulators/Markers” (Figure 6B2), we notice an upregulation between P0 and P2 of the gene *PPARG*, *WISP1,* and *DKK1*, which was compensated by a slight reduction of other differentiation markers such as *CD34*, *CD36*, *CD146, ZFP423,* and *α-KLOTHO*. *RUNX2* is a transcription factor essential for osteoblast differentiation and chondrocyte maturation. This marker is very useful for verifying whether hASCs begin to differentiate spontaneously towards the osteo- or chondro-lineage. In the corresponding heatmap below, the white color and the blue shades prevail over the red colors, so the marker/regulator gene expression’s general trend does not indicate a dramatic change in their expression at P2. Finally, with the third category, the “Lineage Hierarchy Markers” genes, which were recently described by David Merrick et al. [45], their trend is more oriented towards the status quo or a slight increase in the expression of the tested genes at P2 (Figure 6B3). This is graphically highlighted in the heat map below the corresponding bar graph, where white and red shades predominate. Thus, as seen before, with the flow cytometry analysis, we can conclude that it is possible to grow and amplify hASCs up to passage P2 with our *UrSuppe* culture medium without compromising the undifferentiated state of the cells.

### 3.5. hASCs’ Differentiation with Serum- and Xeno-Free Media

#### 3.5.1. hASCs Induced in Defined Conditions to Differentiate into White or Beige Adipocytes

Expansion of hASCs represents only the first step in a cell therapeutic protocol. In fact, before using these cells, it is necessary to induce their maturation/differentiation into a “stable” and specific lineage (e.g., adipocyte, chondrocytes, osteocytes, muscle cells, and so on). This is very important to reduce or avoid the risk of differentiation into an unwanted cell type or the potential risk of stimulating the growth of undetected tumors (bystander tumor formation) [69]. To confirm that hASCs expanded in *UrSuppe* retain their ability of multi-lineage differentiation, we perform a classical tri-lineage differentiation assay using a commercially available kit. Adipogenic differentiation was determined by observing the presence of Oil Red O-stained fat vacuoles in cells. The chondrogenic differentiation was evaluated by Alcian Blue staining and, finally, the osteogenic differentiation capacity was highlighted by Alizarin Red S staining. We found that hASCs expanded in *UrSuppe* medium showed trilineage differentiation capabilities (see Supplementary Information Figure S7 and paragraph S7: Differentiation assay to verify hASC multipotency).

**Figure 7:**
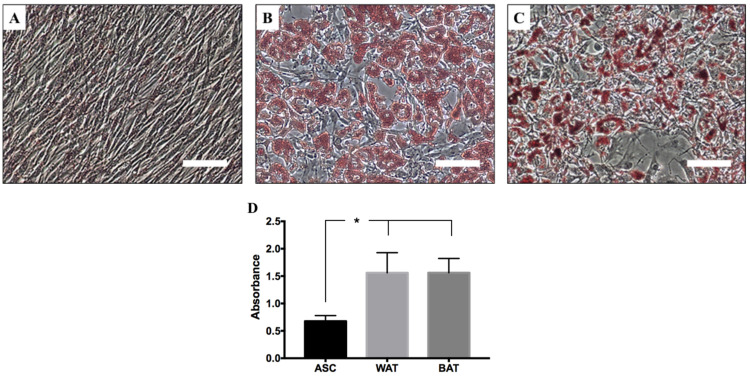
Adipose conversion shown by lipid staining with Oil Red O. Human ASCs at early passage (P3) expanded in *Ursuppe* basal medium are easily induced to differentiate into early adipocytes. Two different xeno- and serum-free induction media developed in our laboratory were used for these assays. Fold magnification 100×. Scale bar 100 µm. (**A**) Not induced hASC, grown on *UrSuppe* basal medium (negative control); (**B**) hASCs after ten days of white adipogenic induction with *UrSuppe*-white adipose tissue (US-WAT) medium; (**C**) hASCs after seven days of beige adipogenic induction with *UrSuppe*-beige/brown adipose tissue (US-BAT) medium. (**D**) Evaluation of the adipogenic differentiation based on the quantification of the Oil Red O absorbance, normalized for the number of cells present in the cell culture vessel. Data represent the mean absorbance ± SEM. To detect significant differences, a one-way analysis of variance (ANOVA) test was performed. * *p*-value < 0.05.

**Figure 8:**
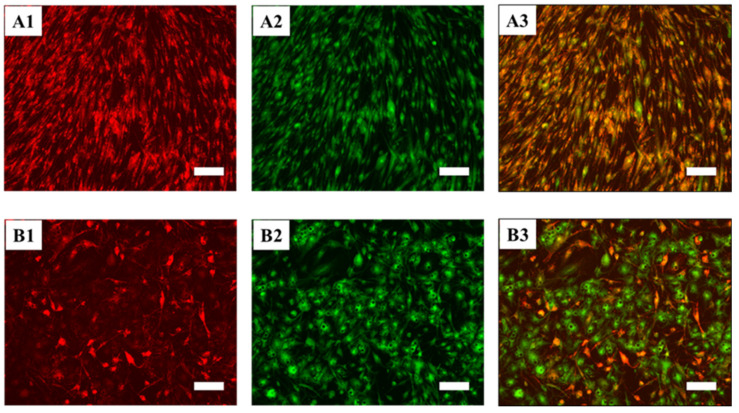
JC-10 staining shows mitochondrial depolarization after culturing confluent hASCs in the US-BAT medium. (**A1**–**A3**) Negative control: not induced hASC, grown on *UrSuppe* basal medium. (**B1**–**B3**) BAT induction, green fluorescence: changes of the membrane potential (caused by UCP1 expression). (**1**) Representative microphotograph taken with a 590 nm filter showing JC-10 aggregates in normal cells. (**2**) Representative microphotograph taken with a 525 nm filter showing JC-10 monomer in depolarized mitochondria. (**3**) Merged. Fold magnification 100×. Scale bar 150 µm.

There are numerous possible applications of adipose tissue in regenerative medicine [70,71,72], and some of them were unexpected and surprising until recently [73]. This without forgetting the interest in finally being able to study the biology of adipocytes in vitro under defined cell culture conditions [19]. As a first step in this direction, we developed xeno- and serum-free media, which induce the maturation of hASCs into white or beige adipocytes and which we named “*UrSuppe*-white adipose tissue” (US-WAT) and “*UrSuppe*-beige/brown adipose tissue” (US-BAT). To evaluate our media’s adipogenic differentiation potential, hASCs were initially seeded and expanded in *UrSuppe* basal medium until confluency was reached. Adipogenic differentiation was then induced with US-WAT medium for ten days or with US-BAT medium for seven days. Human ASCs efficiently differentiated into pre-adipocyte accumulating lipids as visualized by the Oil Red O staining for both media (Figure 7A–C). The dye was extracted from the cells using 2-propanol, and absorbance was photometrically determined to enable quantitative measurements. As shown in Figure 7D, intracellular lipid accumulations are tripled when cells are cultured with either the US-WAT or US-BAT medium. As opposed to white adipocytes, both brown and beige adipocytes possess thermogenic properties and thus can produce high levels of heat via uncoupled respiration. This is achieved by expressing high levels of uncoupling protein 1 (UCP1) to dissipate energy in the form of heat by uncoupling the mitochondrial respiration. This phenomenon, the mitochondrial membrane’s depolarization, can be visualized by the JC-10 molecule [74]. This dye accumulates in mitochondria of healthy cells with polarized mitochondrial membranes, generating an orange emission profile. As the membrane potential decreases, which results in the failure to retain JC-10-aggregates in the mitochondria, JC-10 monomers are generated, resulting in a shift to green emission. As shown in Figure 8, this happens when hASCs are cultured with the induction medium US-BAT.

#### 3.5.2. RT-qPCR Analysis of Induced hASCs

**Figure 9:**
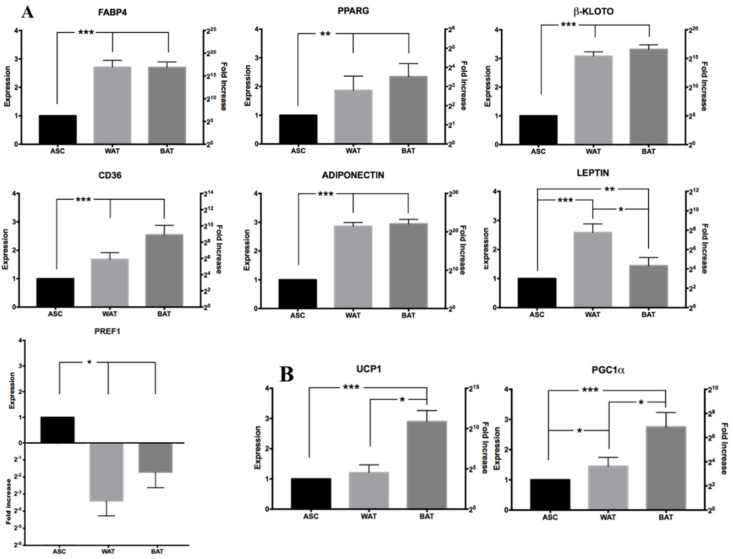
RT-qPCR analysis of different genes involved in WAT and BAT differentiation. (**A**) Expression of seven common genes involved in adipogenesis regulation. (**B**) Expression of the two main common genes involved in the maturation of brown/beige adipose tissue. Data represent the mean fold-increase expression in different medium ± SD versus ASC. Gene expression is normalized to undifferentiated ASC (indicated on the “Expression” *y*-axis), the different expression of genes in WAT or BAT differentiation medium are indicated on the “Fold Increase” *y*-axis on the logarithmic scale. To detect a significant difference, a one-way ANOVA was performed for each gene. * *p*-value < 0.05; ** *p*-value < 0.01 *** *p*-value < 0.001.

To confirm the cells’ correct differentiation, RT-qPCR analysis was performed for selected genes involved in the adipogenic process. Most of them are involved in the formation of lipid droplets and the adipocyte’s metabolic regulation, a process that occurs in white or in beige/brown adipose tissue. Further information about these genes, including a short description and references, can be found in Supplementary Information (Tables S6–S8). As shown in Figure 9, all marker genes linked to adipose-development and maturation are strongly upregulated when hASCs are cultured in both WAT and BAT induction media, without a significant difference between the two inductions. The only gene that was downregulated is *PREF1*. This was expected as this marker plays a crucial role in preserving an undifferentiated status and blocking adipogenic differentiation [75,76]. However, when the cells begin to mature into adipocytes, *PREF1* is drastically downregulated. Interestingly, we noticed that the *LEPTIN* expression levels were statistically significantly higher in the WAT induction than in the BAT induction. This is in line with what is known about this hormone, mainly produced by the WAT and poorly expressed in BAT [77,78]. Finally, Figure 9B shows the expression levels of two of the central genes responsible for the formation of brown/beige adipose tissue, *UCP1* [79] and *PGC1α* [80,81]. Both are detected in hASCs induced with US-WAT or US-BAT media. However, as expected, their expression is significantly higher when the BAT induction medium is used.

#### 3.5.3. Flow Cytometry Analysis of Induced hASCs

In a similar way to what was shown before in paragraph 3.1 with the SVF, we chose 12 reliable cell surface proteins that characterize undifferentiated ASCs or early adipocytes. These markers can be classified as follows:

- Canonical ASCs markers: CD73, CD90, CD105 [27];
- Adipose progenitor cell markers: CD26 (DPP4), CD54 (ICAM-1), CD55 (DAF), CD142 (tissue factor, TF), CD248, Endosialin [45,62,63];
- Adipogenic differentiation markers: CD34, CD36, CD146 [30,33,34,41];
- Beige adipocyte marker: CD137/TNFRSF9 [82,83,84].

**Figure 10:**
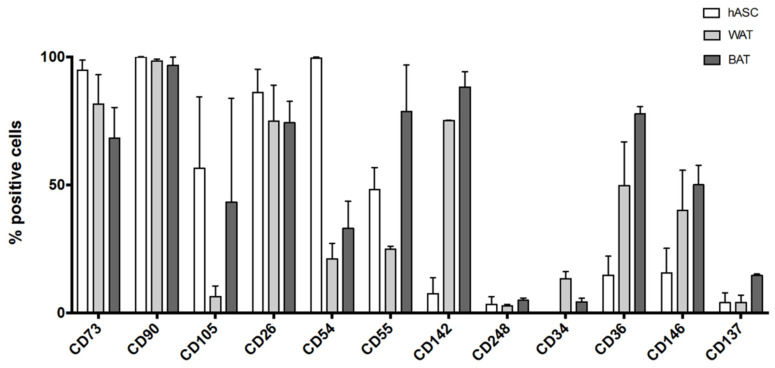
Percentages of cells found to be positive for the 13 surface markers chosen for this analysis. Bar chart of summarized flow cytometry data for hASCs cultured in *UrSuppe* basal medium (white bar) or for cells differentiated in either US-WAT (grey bar) or US-BAT (black bar) medium. The chart shows the mean percentage of positive cells for the indicated surface markers. Average of data obtained from three different donors (n = 3, error bars represent S.E.M.).

Human ASCs harvested from three different donors were either maintained in culture as undifferentiated cells with *UrSuppe* basal medium or differentiated with US-WAT or US-BAT. Before harvesting the cells, the undifferentiated hASCs showed the typical elongated spindle-shaped morphology. In contrast, most induced cells were polygonal with numerous cytoplasmic lipid droplets of different sizes, as shown in Figure 7A–C. To control for changes in surface marker expression due to the different culture media, the cells at passage two were detached from the 60 mm coated Petri dishes, as described in Section 2.1.3., and were analyzed by flow cytometry. As shown in Figure 10, the majority of the hASCs grown in *UrSuppe* basal medium were positive for the “canonical markers” (CD75, CD90, CD105) and for the majority of the recently described “adipose progenitor cell markers” (CD26, CD54, CD55). We noticed that the percentage of CD105 positive hASCs was lower than the two other “canonical markers”. However, this phenomenon was already reported in other studies, where cells were grown in serum-free conditions [85,86]. Two surface proteins of the group “adipogenic differentiation markers”, CD142 and CD248, were only detected on a small percentage of the hASCs. CD142 was described by Schwalie et al. [62] as a marker of a small subpopulation of early precursors, which can suppress adipocyte formation in a paracrine manner. Thus, the fact that CD142 is expressed by progenitor cells with a regulatory task makes it reasonable only to expect its presence on the surface of a small percentage of hASCs. CD248 was identified by Merrick et al. [45] after single-cell RNA sequencing and cell trajectory analyses and classified as one of few markers to be expressed by very early adipose progenitor cells. However, new results have shown that CD248 expression could mark malfunctioning cells [66]. Given this information, it is very positive that only a small percentage of the hASCs grown in *UrSuppe* basal medium are CD248 positive. Likewise, positive percentages for CD34, CD36, and CD146 remain low when culturing hASCs with the *UrSuppe* medium. In contrast, the percentages of positive cells for the “adipogenic differentiation markers” clearly increased when hASCs were differentiated with US-WAT or US-BAT medium. In parallel, some markers of the undifferentiated status, such as CD54, CD55, and CD105, show a strong decrease in positive cells. Finally, the number of CD137 positive cells clearly increased only when the hASCs were induced with US-BAT medium. Taken together, these data suggest that it is possible to grow hASCs under xeno- and serum free conditions while preserving their undifferentiated status. This analysis also revealed that the differentiation media were effective by boosting the expression of the three “adipogenic differentiation markers” and of CD137 with US-BAT.

#### 3.5.4. Comparative Secretome Profile Analysis of Undifferentiated and Differentiated hASCs

**Figure 11:**
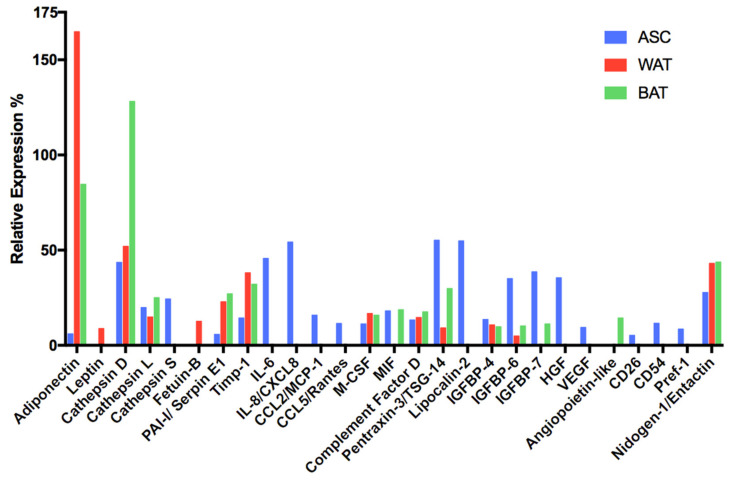
Proteome profiler of hASCs grown in *UrSuppe* medium (blue bar), US-WAT (red bar), or US-BAT (green bar) induction medium. Data are represented as mean pixel intensity (MPI) of fluorescence measured by the Licor acquisition system. Measured values were normalized on background fluorescence and on positive control to obtain a relative MPI. This experiment was performed with hASCs obtained from the biopsy of one donor. The following factors are not shown in the graph because of a very low expression in undifferentiated hASCS: *Chemerin, Fibrinogen, IGFBP-2, LIF, PAPP-A, PCSK9, Aerpin A8*.

Because some polypeptides are only produced and secreted during certain precise stages of development or maturation, they can be used to diagnose a specific metabolic state or a specific differentiation degree of the cells under investigation. To assess 58 human adipokines’ presence, we used a commercially available “Proteome Profiler Human Adipokine Array” (Figure 11, and Supplementary Material Figure S8 and Tables S9 and S10). Interpreting and finding a biological sense for a proteome profiler array is always very difficult, especially if many polypeptides are detected. In this case, we notice some trends that facilitate interpreting the obtained results.

First, the detected polypeptides were already associated by other reports to the adipose tissue’s secretome (for review, see [87]). Second, to facilitate the data’s comprehension, it was possible to group the secreted polypeptides according to some characteristic traits that unite them. The three profiles are different, as can be seen by looking at Figure 11 and Table S9.

As a first very positive result, we found that two signature adipokines (adiponectin and leptin, reviewed in [88]) for differentiated cells were very low or missing in the supernatant of non-induced hASCs, while present in cells cultured with the WAT or BAT differentiation medium. Interestingly, it is known that leptin is secreted mostly by mature subcutaneous white adipocytes [89], and indeed, this is what we observed in this assay and by the RT-qPCR measurements shown in Figure 9.

Next, we noticed the presence of three Cathepsin family members. These proteins are proteases that play different crucial roles in mammalian cellular turnover. Cathepsin D [90] (aspartyl protease) and Cathepsin L [91] (cysteine protease), as well as Cathepsin S (cysteine protease), play essential physiological roles in the adipose tissue and are essential for remodeling the extracellular matrix (ECM) [92]. Interestingly, Cathepsin D also has mitogenic activity independent of its proteolytic activity, and its knock-out in mice revealed that it is indispensable for postnatal tissue homeostasis [93]. Together with the proteases, we detected three protease inhibitors (Fetuin-B, PAI-I, Timp-1). Among these three proteins, the most exciting factor is Timp-1 (tissue inhibitor of metalloproteases). This inhibitor orchestrates with the matrix metalloproteases (MMP) the regulated turnover of the ECM, and its role is to control the proteases’ enzymatic activity [94]. Surprisingly, it was also discovered later that TIMPs act as signaling molecules with cytokine-like activities, thus influencing various biological processes, including cell growth, apoptosis, differentiation, angiogenesis, and oncogenesis. All these functions are independent of MMP inhibition [95,96].

The proteome profiler array also highlighted several cytokines [97] and chemokines [98] in the cell-conditioned culture media: IL-6, IL-8, CCL2/MCP-1, CCL5/Rantes, M-CSF, MIF, complement factor D, Pentraxin-3/TSG-14, and Lipocalin-2. It is challenging to find a common denominator for these secreted factors, mostly associated with an inflammatory condition. However, it has been reported recently that proinflammatory signaling in the adipocyte is required for proper adipose tissue remodeling and expansion [99,100]. Human ASCs in culture are likely to secrete these cytokines and chemokines to form the best environment around them to grow and proliferate. It is well known that, in the adipose tissue, numerous adipocyte-derived adipokines involved in modulating metabolic homeostasis also display immunomodulatory properties. For example, we can mention the pleiotropic cytokine IL-6, which was attributed to an important role in the “browning” of the white adipose tissue [101]. The last factor in this group is Lipocalin-2, which has been attributed to the innate immunity to sequester iron and prevent its use by bacteria, thus limiting their growth. However, Lipocalin-2 may preserve adipose tissue function and protects against age-related metabolic misfunctions. Interestingly, this factor was found only in the supernatant of non-induced hASCs [102].

The next group of related polypeptides consists of the insulin-like growth factor-binding proteins (IGFBPs). One of their primary functions is to regulate the action of insulin-like growth factors (IGF-I, IGF-II) by controlling the accessibility of these peptide hormones to their receptors [103]. Both IGF-I [104] and IGF-II [105,106] are pro-adipogenic. It has been shown that IGFBP-4 binds IGF-I and its expression negatively correlates with adipose tissue growth [107]. IGFBP-6 can bind IGF-II, and IGFBP-7 can bind both IGF-I and II [108]. Thus, it is likely that these three members of the IGFBP family are anti-adipogenic. In fact, the general tendency is to decrease when the cells are induced to differentiate. The most intriguing member of this group of secreted factors is IGFBP-7, also known as Angiomodulin (AGM), and reported to be expressed by the developing vasculature [109]. It binds chemokines and growth factors, including VEGF-A [110,111]. Therefore, IGFBP-7 is now considered proangiogenic in the developing vasculature, that is, in concert with VEGF-A and potentially other angiogenic factors, it guides and stabilizes the nascent vasculature. Interestingly, IGFBP-7 and VEGF (reviewed in [112]) are simultaneously found only in the supernatant of uninduced hASCs. Two other polypeptides related to angiogenesis and cell proliferation were detected: hepatocyte growth factor (HGF, reviewed in [113]) and angiopoietin-like factor [114].

We detected the soluble version of CD26, CD54, and Pref-1 as hASC-specific secretions. These proteins are encoded by genes classified as adipose progenitor cell markers. Therefore, we consider their exclusive presence in the conditioned medium of undifferentiated cells as a positive result that confirms the data obtained previously by flow cytometry or RT-qPCR.

Finally, Nidogen/Entactin is a typical component of the ECM found in the adipose tissue [94] and, therefore, it is not surprising to find it in the cell supernatants.

In conclusion, the secretion profile of hASCs is different from that obtained with cells induced to differentiate into white or beige adipocytes. Some typical adipokines that signal ongoing adipogenesis, such as Adiponectin and Leptin, are very low or completely missing. Conversely, some factors known to be anti-adipogenic, or angiogenic, or related to progenitor cells are mainly present in the supernatant of non-induced hASCs. Thus, we can cultivate hASCs with the *UrSuppe* medium while maintaining their undifferentiated status.

### 3.6. Is Human Platelet Lysate the Best Possible Substitute for FBS?

**Figure 12:**
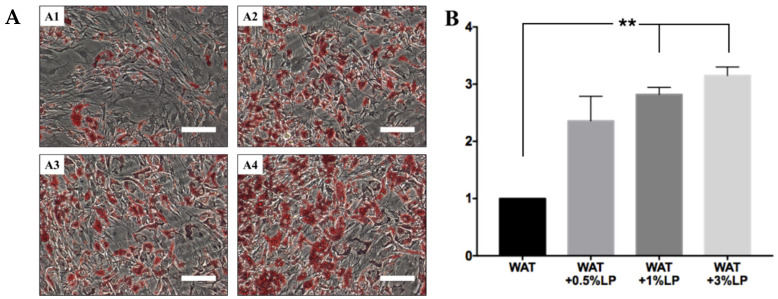
Oil Red O staining and quantification to evaluate the effect of hPL on adipogenic differentiation. hASCs after 10 days of adipogenic induction with or without hPL supplement (0.5%, 1%, 3%). (**A**) Representative phase-contrast microphotographs illustrating triglycerides accumulation: (**A1**) US-WAT only, (**A2**) US-WAT + 0.5% hPL, (**A3**) US-WAT + 1% hPL, and (**A4**) US-WAT + 3% hPL. Fold magnification 100×. Scale bar 100 µm. (**B**) Quantification of Oil Red O staining. 100% 2-propanol was used as background control and subtracted to the four test samples’ measured optical densities (ODs), which were then normalized on the number of nuclei present on every dish. The data refer to the value obtained with cells grown with US-WAT only, thus this bar chart shows the fold variation of absorbance related to this sample. The results represent the average ± SD of three different samples. Statistical analyses were performed as one-way ANOVA using Prism7 software, ** *p* < 0.01.

In these last years, the use of FBS and other animal derivatives for ex vivo expansion of human cells has been discouraged by regulatory authorities to reduce the risk of transmitting prions and other zoonoses and avoiding xenogeneic immune reactions in the host. Consequently, the authorities demand important scientific justifications if one wants to use FBS for the cell culture process, as well as numerous specific analyses demonstrating the absence of potentially dangerous contaminants, such as viruses, prions, and endotoxins [115]. Faced with these difficulties, it is not surprising that researchers have developed human alternatives to FBS for cell expansion. Human platelet lysate (hPL) is currently considered the ideal substitute for FBS and is regarded as an acceptable alternative by the regulatory authorities [116]. However, the use of hPL presents some disadvantages [117]. Indeed, similarly to FBS, it is not precisely defined, and this implies that hPL may contain growth factors or hormones that can induce spontaneous differentiation of the cultured progenitor cells. Several years ago, it was demonstrated that sera of different species, including human, contain an “adipogenic factor”, which induces adipose conversion in undifferentiated cells. Using the same strategy applied by Walid Kuri-Harcuch and Howard Green [118], we wanted to determine whether hPL also contains pro-adipogenic activities. For this purpose, hASCs at passage 2 were cultured in 60 mm Petri dishes in *UrSuppe* basal medium until confluency. At this point, the cell culture medium was changed to one based on the adipogenic induction medium US-WAT supplemented with 0.5%, 1%, or 3% hPL. The control test received US-WAT medium without hPL supplement. As shown in Figure 12A, supplementing US-WAT with hPL strongly boosted the conversion of hASCs into adipocytes, as evidenced by the increasing number of fat vesicles stained with Oil Red O. This dye was then extracted from the stained samples with 2-propanol, and the optical density (OD) was read with a photometer. Figure 12B confirms increased lipid storage in those samples where US-WAT was supplemented with 0.5%, 1%, or 3% hPL. This rise in measured triglycerides is not due to increased cell numbers because the data were normalized on every dish’s number of nuclei. We concluded that hPL, similarly to all the other undefined sera used in cell culture, contains large amounts of pro-adipogenic factors. This is not a surprise, as proteomics studies completed in recent years on the composition of hPL have revealed the presence of numerous factors, e.g., IGF-1 and several members of the bone morphogenetic proteins (BMPs) family [119,120]. These proteins play essential roles in the induction of adipogenesis and/or chondrogenesis, respectively. Taken together, we conclude that hPL may not be the best choice to replace FBS in cell culture because it does not rule out a spontaneous, undesired slow differentiation of the cultured undifferentiated cells.

## 4. Discussion

Adipose tissue is remarkably understudied compared with other organs. Until the mid-20th century, it was simply considered as a metabolically inert connective tissue with no particular functional properties, except the ability to store excess energy in the form of intracellular triglycerides. However, in recent years, owing to increasing obesity and obesity-associated pathologies globally, this tissue has gained significant attention from the research community. Now, it is recognized as a proper active endocrine organ at the center of energy balance and nutritional homeostasis, mediating the regulation of multiple organs and tissues [1,2,121]. Finally, many groups have shown independently that the adipose tissue is particularly rich in adult stem cells, which can differentiate, along multiple pathways, raising great hope in regenerative medicine [72,122]. A striking aspect of adipose tissue is the extreme complexity of its developmental biology. Recent studies suggest that anatomically distinct white adipose tissue depots originate from different precursors that are destined to become subcutaneous or visceral adipocytes [3,21,123]. Furthermore, adipose depots’ initial development during the fetal and early postnatal period depends on precursors distinct from those that contribute to tissue maintenance and remodeling in adulthood [124,125]. All of this probably leads to the intra-depot heterogeneity recently documented in the adipose tissue [20,126]. This extraordinary complexity is also reflected in the fact that the precise anatomical location in the adipose tissue of these progenitor cells is still unclear. Many reports indicate a perivascular localization of progenitor cells, suggesting a mural cell origin of adipogenesis. However, other recent studies revealed non-perivascular adipose precursor populations [45,127]. Thus, it is not surprising that this diversity has led to the characterization of various cell types believed to be WAT progenitors (reviewed in [128]).

Initially, Rodeheffer et al. identified and Berry et al. characterized a subpopulation of early adipocyte progenitors defined as CD24^+^ CD29^+^ CD34^+^ Sca-1 (Ly6A)^+^ in mouse WAT [31,129]. Since then, many studies have found distinct adipocyte progenitor cells with various cell surface proteins expressed in WAT [128] and, recently, single-cell RNA sequencing (scRNAseq) technology was used to find new gene markers able to better define the cellular subpopulation involved in adipogenesis (reviewed in [130,131]). This new way to search for new markers led David Merrick et al. [45] to identify distinct types of progenitor cells in murine subcutaneous adipose tissue, which were subdivided into three hierarchical groups based on their gene expression patterns (reviewed in [132,133]). The first population, termed “interstitial progenitor cells” (IPCs), is highly proliferative and undifferentiated. These cells are CD24^−^ CD26^+^ CD34^+^ CD54^−^ CD55^+^ CD142^−^ and are localized in the “reticular interstitium”, a recently described anatomically distinct structure that encases many organs [134]. IPCs then give rise to the two other subpopulations, which are intercalated between mature adipocytes. So, in the mouse, group two cells are defined as CD24^−^ CD26^−^ CD34^+^ CD54^+^ CD55^−^ CD142^−^, whereas group three cells are defined as CD24^−^ CD26^−^ CD34^+^ CD54^+^ CD55^−^ CD142^+^. Similarly, Merrick et al. found for human subcutaneous adipose tissue a gene expression profile for group 1 and 2 similar to that of mouse, while group three could not be assigned because, in humans, CD142 is abundantly expressed both in group 1 and 2. So, the human IPCs cells (group one) are defined as CD24^−^ CD26^+^ CD34^+^ CD54^+^ CD55^+^ CD142^+^ CD248^+^, whereas group two cells are defined as CD24^−^ CD26^−^ CD34^+^ CD54^+^ CD55^−^ CD142^+^ CD248^−^.

The results obtained thanks to scRNAseq technology and published recently by a few research groups [45,62,63], reviewed in [130], are very important and cannot be ignored. Therefore, we selected five of these new markers (i.e., CD26, CD54, CD55, CD142, and CD248), along with five canonicals (CD34, CD73, CD90, CD105, and CD146), to investigate the cells grown in *UrSuppe* medium. As shown in Figure 10, nearly 100% of the cells are positive for CD26, CD54, CD73, CD90, and CD105, while about 50% of them are positive for CD55. Simultaneously, only small percentages are positive for the differentiation markers CD34, CD36, and CD146. This seems to correspond to a markers profile compatible with undifferentiated cells, which may be described as CD24^−^ CD26^+^ CD34^−^ CD54^+^ CD55^+/−^ CD142^−^ CD248^−^ (as well as CD73^+^, CD90^+^, CD105^+^). Therefore, concerning the classification suggested by Merrick et al. [45], these cells could be classified as an intermediate form between groups one and two. Their differentiation with the induction media US-WAT or US-BAT gave interesting results. As expected, adipogenic markers increased, while only CD54 and partially CD55 (only with US-WAT) and CD105 decreased significantly among the progenitor cell markers. We were surprised that CD26 remained well expressed even when the cells were cultured with the induction media. Flow cytometry is notoriously difficult to apply to investigate pre- or mature adipocyte cell surface markers expression [108] because the cells are very large, fragile, and have high buoyancy. Thus, many of them are damaged or lost during the samples’ preparation, and less differentiated cells have a better chance of being analyzed. We also remarked that CD26 is present on many types of cells of the SVF and found in combination with CD36, which is a marker associated with adipogenesis [40,41] (Figure 1B). This suggests that this surface protein is not strictly linked with progenitors, but can also be expressed by committed or more mature cells. This could reduce its usefulness as a tool for selecting cells with a high degree of “stemness”.

Another exciting marker used in our study is CD142. This surface protein was associated with progenitor cells named A_reg_, capable of negatively regulating adipogenesis [62]. However, we found during our study that Dexamethasone strongly induces CD142, a synthetic corticosteroid typically used in induction media for its strong pro-adipogenic effects [135,136] (Figure 10 and Supplementary Materials Figure S9). Thus, it is difficult to reconcile this observation with the claim that CD142 labels cells with anti-adipogenic potentials when an adipogenesis-promoting hormone induces its expression. This perplexity is also shared by Merrick et al., who found CD142^+^ cells to be highly adipogenic both in vitro and upon transplantation in vivo [45]. In 2008, the first study describing a population of progenitor cells postulated the existence of inhibitory cells in the WAT [31]. Petra Schwalie et al. have again drawn attention to this aspect, but further studies will be needed to complete Areg’s characterization and to understand the role of CD142 in this context [62].

Recently, again thanks to the scRNAseq technology, Ogury et al. reported the characterization of the CD81 surface protein as a marker of beige adipocyte precursor cells, which give rise to thermogenic adipocytes in subcutaneous adipose tissue [137]. We tested this marker in the context of the flow cytometry platform we usually use to characterize the cells of the SVF. As shown in Supplementary Materials Figure S10A, CD81 is not a rare antigen and is present in the three main subgroups with which we divide the cells of the SVF (i.e., CD34^+^CD146^−^, CD34^-^CD146^+^, and CD34^+^CD146^+^). Many of these cells are also double positive for CD36, a notorious marker signaling adipogenic differentiation. We have consistently observed that the CD34+CD36+ subpopulation cells display the highest CD81 mean fluorescence compared with the other two subgroups (Supplementary Materials Figure S10B). Finally, this marker is abundantly expressed by almost the totality of the hASCs grown and expanded with the *UrSuppe* cell culture medium (Supplementary Materials Figure S10C). CD81 belongs to the Tetraspanins, a large family of transmembrane proteins ubiquitously expressed and implicated in cell proliferation, differentiation, and tumor invasion [138,139]. Thus, this marker is not exclusively expressed on beige adipocyte precursors, but also by highly proliferative ASCs [137]. Taken together with these properties, it will be difficult for the CD81 marker to find practical applications by itself, such as characterizing and/or purifying beige adipose progenitors from human adipose tissue.

**Figure 13:**
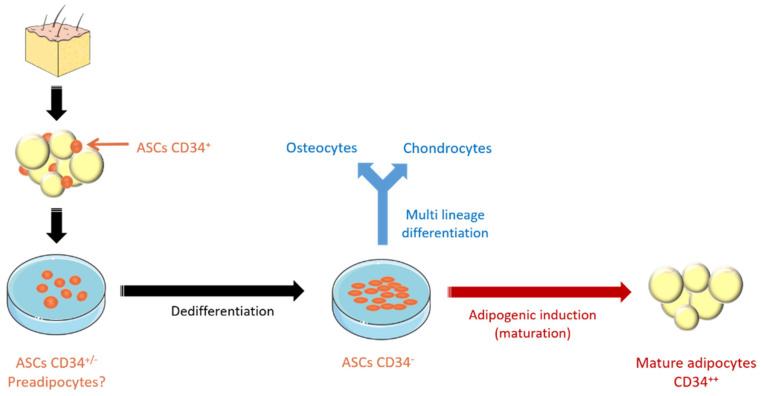
Preadipocytes’ dedifferentiation induced by the ex vivo cell culture conditions.

The transmembrane phosphoglycoprotein CD34 was first identified as a hematopoietic stem cell marker, but was later found on several nonhematopoietic cell types [140]. In the clinic, this marker is associated with the selection and enrichment of stem cells for bone marrow transplants. Therefore, because of these historical and clinical associations, CD34 has always been linked with “stemness” in various systems, including hASCs. Freshly extracted hASCs are CD34^+^ (Figure 1B), but they lose this marker upon plastic adhesion and culture (Figure 5 and Figure 10). This change in hASCs’ phenotype has been considered as a differentiation process in response to the in vitro environmental changes. However, various published studies and our direct observations suggested a different interpretation to explain this phenomenon. Indeed, CD34 is expressed on the surface of mature adipocytes [30] and is upregulated when hASCs are induced to mature toward an adipogenic lineage using a differentiation cell culture medium (Figure 10) [33]. These data might suggest that CD34, at least in the adipose tissue, does not signal “stemness”, but rather a marker of adipogenic differentiation expressed by early and mature adipocytes. Therefore, the decrease in CD34 during the early stages of in vitro culture might signify that the cells dedifferentiate and thus increase their “stemness”. This hypothesis might be plausible given that the culture conditions are known to change the fate and potency of the growing cells [141]. Furthermore, adipocytes have been shown to possess a high degree of plasticity and can switch from a mature form to a less differentiated one [142]. All these considerations are depicted in Figure 13. Finally, these thoughts led us to suspect that the hASCs grown in *UrSuppe* medium may perhaps be placed in a hierarchical line upstream to the IPCs described by Merrick et al. Single-cell RNA sequencing technology applied to hASCs cultured according to our conditions may help us to understand whether this hypothesis is correct or wrong.

Expanding undifferentiated cells in vitro without affecting their “stemness” is very important. To achieve this, we developed a newly defined xeno- and serum-free medium, called *UrSuppe*, specially formulated to grow hASCs. Studies focusing on hASCs grown in serum-free media are relatively rare, and in Supplementary Materials Table S11, we listed a good portion of them with some basic information and references. Using the *UrSuppe* cell culture medium, we achieved specific growth rates for hASCs isolated from different patients (i.e., age, health condition) comparable to those reported by other researchers [58,59,143]. The maximum achieved cell densities were in a clinically significant range, especially if the expansion processes are transferred to larger cultivation systems (e.g., stirred single-use bioreactors in combination with microcarriers) in the future. FBS is still the most used cell culture medium supplement, although it is an undefined cocktail of numerous growth factors and hormones [144,145]. However, it is very difficult to seal the “stemness” of the expanded hASCs with this component. Indeed, it was shown a long time ago that FBS and other sera, including human, contain adipogenesis promoting factors [118]. The same applies to the platelet lysate (PL); despite being produced from human blood, it is a cocktail of numerous growth factors and hormones, some of which have well-known adipogenic promoting activity [116,120] (Figure 12), which acts in an unpredictable and uncontrolled way on the fate of the hASCs [146,147].

For these reasons, this study’s main objective was to develop a chemically defined cell culture medium containing specific components capable of supporting the growth of hASCs while preserving their undifferentiated status. The *UrSuppe* medium is specific for the cultivation of hASC and contains only recombinant growth factors and synthetic hormones, which enhance the stemness, and is devoid of all known components that could induce cell differentiation. To be consistent with the approach used to expand hASCs, we also developed xeno- and serum-free (XFS) media to induce the maturation into white or beige adipocytes. The fascination with adipose tissue has increased enormously because of the worldwide escalation of obesity and related metabolic diseases. Therefore, it will also be necessary to have cell models obtained under defined conditions to study in vitro the adipocyte biology in health and disease.

The analytical results obtained during this study from hASCs cultured in *UrSuppe* medium confirmed that the cells preserved an undifferentiated status and specific stem characteristics. Typically, a well performing defined XSF medium, like *UrSuppe*, is unique for the type of cell for which it was explicitly developed. For this reason, it is hardly suitable for culturing other cell types [148,149]. This specificity linked to the type of cell of interest can be considered an intrinsic disadvantage of defined XSF media. Changing the cell type often forces the researcher to develop a new type of XSF medium. This task is not always easy and can take time. However, we think that the advantages of developing this crucial ancillary material for the cell culture are as follows:

- It is possible to improve/adapt the formula continuously.
- It facilitates subsequent steps (e.g., differentiation into mature lineages with specific XSF induction media).
- It provides better access to troubleshooting.
- It provides better control over the cell culture conditions and thus over the whole process.

Aside from the considerations, there are the classic motivations for excluding hPL (reviewed in [117]) and FBS (reviewed in [148,150]) from the cell culture medium used to grow therapeutic hASCs; indeed, when FBS is used, the risk of transferring infectious agents from animals cannot be eliminated. Besides, FBS is engulfed by the stem cells and may induce immune xenogenic rejection upon autologous transplantation into the human patient [151]. Furthermore, except for blood and endothelial cells, all the other tissue cells usually never come into contact with serum. They are in contact with the interstitial fluid, which has a lower concentration of proteins than serum, and is specific for each tissue or organ owing to a unique composition of secreted polypeptides and metabolic molecules [152,153], which is thus a situation we can try to reproduce and mimic in serum-free conditions. Finally, there are also serious ethical concerns about FBS’s use concerning its harvesting and production process [154], not to mention the risk of adulterated composition and unstable prices. All these are good reasons to move away from undefined components in general and FBS in particular.

## Figures and Tables

**Table 1:** Overview of donor-related data. The passage number and population doubling level (PDL) refer to the cell inoculum used for the growth characterization.

Donor	Gender	Age	Region	Health Stats	P_inoculated_	PDL_inoculated_
D-I	Female	46	Abdomen	Healthy	P2	6.3
D-II	Female	26	Abdomen	Post-chemotherapy	P2	6.5
D-III	Female	50	Abdomen	Post-chemotherapy	P2	6.0
D-IV	Female	50	Abdomen	Healthy	P3	8.1

**Table 2:** Overview of the main growth-dependent parameters in the 2D cultivations (n = 2 per donor).

No.	X_max_	PDL *	EF **	µ	t_d_	Y_Lac/Glc_	q_Glc_	q_Lac_	q_Amn_
(-)	(10^5^cells/cm^2^)	(-)	(-)	(d^−1^)	(h)	(mmol/mmol)	(pmol/cell/d)		
D-I	0.76 ± 0.05	2.9 ± 0.1	7.6 ± 0.7	0.45 ± 0.01	36.7 ± 0.5	1.41 ± 0.16	0.30 ± 0.10	0.43 ± 0.05	0.09 ± 0.04
D-II	0.51 ± 0.03	2.4 ± 0.1	5.1 ± 0.4	0.36 ± 0.02	45.6 ± 2.3	1.47 ± 0.21	0.65 ± 0.08	0.95 ± 0.01	0.16 ± 0.03
D-III	0.80 ± 0.09	3.0 ± 0.2	8.0 ± 1.2	0.30 ± 0.03	59.7 ± 6.8	1.60 ± 0.11	0.74 ± 0.10	1.19 ± 0.10	0.26 ± 0.04
D-IV	0.68 ± 0.08	2.8 ± 0.2	6.8 ± 1.1	0.38 ± 0.02	43.8 ± 2.7	1.22 ± 0.11	0.50 ± 0.05	0.61 ± 0.06	0.14 ± 0.01

* Value was calculated based on the values of the stationary growth phase (mean ± σcells). ** Value was calculated based on X_inoc_ and X_max_. X_max_: maximum cell concentration on the planar growth surface. PDL: population doubling time. *EF*: expansion factor. µ: specific cell growth rate. td: doubling time of cell population. Y_Lac/Glc_: lactate yield per glucose equivalent. q_Glc_: specific glucose consumption rate. q_Lac_: specific lactate pro-duction rate (growth-dependent). q_Amn_: specific ammonium production rate (growth-dependent).

## Supplementary Materials

The following are available online at https://www.mdpi.com/2073-4409/10/2/466/s1, Figure S1: Description of the dispensing connector used for the transfer of the liposuction; Figure S2: Description of the equipment used to extract the SVF from 50 mL human adipose tissue; Figure S3: Diagram depicting the steps following the digestion with the Collagenase; Figure S4: Boxplot representing mean and 95% confidence interval of the number of total cells and ASCs; Figure S5: ASCs were grown in SF conditions; Figure S6: Controlled cooling rate protocol for the cryopreservation of freshly extracted cells of the SVF; Figure S7: Multipotency assay of hASCs; Figure S8: Comparing the secretion profile of hASCs; Figure S9: Representative flow cytometry expression of CD142 after Dexamethasone stimulation; Figure S10: Representative flow cytometry distribution of CD81 in the cells of the SVF and during culture. Table S1: Materials; Table S2: Detailed information of the antibodies used for the flow cytometric measurements; Table S3: Reverse transcription detailed procedure; Table S4: Primers sequences; Table S5: RT-qPCR cycle conditions; Table S6: Overview of measured stemness maintenance genes; Table S7: Overview of measured differentiation regulators/markers; Table S8: Overview of measured lineage hierarchy markers; Table S9: Qualitative evaluation of the factors secreted by hASCs; Table S10: Human adipokine array; Table S11: List of different serum- and xeno-free media used to grow hASCs.

## Abbreviations

**Latin Symbols**		
C_met_	(mmol/L)	Substrate or metabolite concentration
e	(-)	Euler’s number (2.71828…)
EF	(-)	Expansion factor
ΔGlc	(mmol)	Difference in glucose concentration (glucose interval)
ΔLac	(mmol)	Difference in lactate concentration (lactate interval)
P	(-)	Passage number
PDL	(-)	Population doubling level
q_met_	(pmol/cell/d)	Specific substrate consumption or metabolite production rate
t	(d)	Time
t_d_	(d)	Doubling time
Δt	(d)	Time interval
X_A(0)_	(cells/cm^2^)	Cell concentration on planar growth surface at time point 0
X_A(t)_	(cells/cm^2^)	Cell concentration on planar growth surface at time point t
X_A(tmax)_	(cells/cm^2^)	Cell concentration on planar growth surface at the end of the cultivation
Y_Lac/Glc_	(mmol/mmol)	Lactate yield per glucose equivalent
**Greek Symbols**		
μ	(d^−1^)	Specific growth rate
