# Supplementary material for: Chemically Defined Xeno- and Serum-Free Cell Culture Medium to Grow Human Adipose Stem Cells
Source: Cells. 2021 Feb 22;10(2):466. doi: 10.3390/cells10020466 (PMC7926673; doi:10.3390/cells10020466)
Supplement: Supplementary file 1 [file cells-10-00466-s001.pdf]

cells MDPI

Supplementary Information for Materials and Methods Section

Table S1. Materials

Name	# Catalog	Company
3-methyl-1-butanol (isoamyl-alcohol)	412721000	Acros Organics
Phenol/Tris saturated solution	327125000	Acros Organics
JC-10	AG-CR1-3600-M001	Adipogen
Omnifix 100mL Syringe	4614003F	BBraun
CytoFLEX Daily QC Fluorospheres	B53230	Beckman & Coulter
VersaComp Antibody Capture Bead Kit	B22804	Beckman & Coulter
VersaLyse Lysing Solution	B59266AA	Beckman & Coulter
Cell Strainers 40 µm	352340	Becton Dickinson
Cell Strainers 100 µm	352360	Becton Dickinson
Dulbecco's PBS (Ca^{2+} Mg^{2+})	3-05F00-I	BioConcept
Sso Advanced Universal SYBR Green Supermix	1725271	Biorad
Dulbecco's PBS (without Ca^{2+} Mg^{2+})	L0615-500	Biowest
Via1-Cassette (for NucleoCounter NC200)	941-0012	ChemoMetec
Albumin CSL 20%	22918180119611	CLS Behring
Privigen Immunoglobulin	44206-437-05	CLS Behring
Gosselin™ Containers with Snap Cap	PC1000-05	Corning
PureCoat™ Fibronectin Peptide 75cm² Flask	356242	Corning
BioCoat™ Fibronectin-coated Plates	354402	Corning
Screen Matrix	S1001	Denovo Matrix
Cell Counting Kit 8	CK04	Dojindo Laboratories
Trypsin-EDTA Solution	L0930-100	Dominique Dutscher
ED Eppendorf Tubes® 5.0 mL	0030119380	Eppendorf
CTS™ Synth-a-Freeze™ Medium	A13713-01	Gibco
60mm Cell Culture Dish (Col. I coated)	628950	Greiner Bio-One
Syringe Filters	FPE-204-030	Jet Biofil
IRDye®800CW Streptavidin	926-32230	Li-Cor
Accugene™ Molecular Biology Water	51200	Lonza
Nucleospin RNA kit	740955.250	Macherey-Nagel
Hoechst	33342	Molecular Probes
Biobanking and Cell Culture Cryogenic Tubes	368632	Nunc
GoScript Reverse Transcription System	A5001	Promega
Proteome Profiler Human Adipokine Array Kit	ARY024	R&D Systems
Glucose Bio (for Cedex Bio)	06 343 732 001	Roche
Lactate Bio (for Cedex Bio)	06 343 759 001	Roche
NH3 Bio (for Cedex Bio)	06 343 775 001	Roche
Chloroform	C2432	Sigma-Aldrich
Ethanol	51976	Sigma-Aldrich
Fibronectin-like Engineered Protein Polymer	F5022	Sigma-Aldrich

Formaldehyde	47608	Sigma-Aldrich
Glutaraldehyde	G6257	Sigma-Aldrich
Isopropyl Alcohol	348663	Sigma-Aldrich
Oil-Red-O	75087	Sigma-Aldrich
SDS	74255	Sigma-Aldrich
Sodium Chloride	S7653	Sigma-Aldrich
Urea	U5378	Sigma-Aldrich
T_{25} flask	CLS430639	Sigma Aldrich
T_{75} flask	CLS430720	Sigma-Aldrich
Trypan Blue	15250-061	Thermo Fisher
TrypLE Select	12563-029	Thermo Fisher
96 Well Tissue Culture Test Plate	92696	TPP
Cell Spatula	99010	TPP
Collagenase Type B AOF	CLSAFA	Worthington Biochemical Corp.

S1. Isolation of the Stromal Vascular Fraction (SVF) from human adipose tissue

When the subcutaneous adipose tissue was supplied as a whole thick slice, the first operation was to remove the skin using sterile surgical devices, such as scissors, scalpels, and tweezers. The fat tissue was then washed twicewith DPBS supplemented with Ca2+ and Mg2+ (# 3-05F00-I, BioConcept, Switzerland) in a sterile container (Pot Conique PP 1000 mL 105 x 130 mm Couvercle Sterile, # 080866, Milian AG, Switzerland) and cut into small pieces. Subsequently, the tissue was completely disrupted by an immersion blender, and then the homogenized fat tissue was poured into the 100 mL "extraction syringe" (Omnifix 100mL Syringe, BBraun, Melsungen, Germany). For the homogenization of a small amount of fat tissue, the "ULTRA-TURRAX® Tube Drive System" with its single-use and gamma-sterilized DT-50 tubes (both manufactured by IKA-Werke GmbH & Co. KG, Germany; www.ika.com/en) was found to be very practical. On the other hand, if the adipose tissue was collected during liposuction and was already in a "sampling syringe" used by the surgeon, then it could be quickly and safely transferred to the 100 mL "extraction syringe" using a "Fluid Dispensing Connector" (# 415080, FDC1000, BBraun AG, Melsungen, Germany) as depicted in Figure S1. Indeed, our protocol was based on the utilization of a 100 mL "extraction syringe" (Omnifix 100 mL with Luer Adaptor, and Universal Closing Stopper, #4495101, both from BBraun AG, Melsungen, Germany) as a separatory funnel to exploit the fact that the adipose tissue and the hydrophilic fluid containing the cells spontaneously separates in two phases without the need for centrifugation. The piston of the syringe was used to take in or expel the solutions used to wash the sample, dissociate the suctioned fat (Collagenase), or extract the cells from the dissociated adipose tissue. The syringe was held in a vertical position using a laboratory support stand with support rings (Figure S2). Therefore, the syringe allowed all the necessary manipulations to be performed to extract hASCs from 50 mL homogenized fat or lipoaspirate. The procedure took about 70 minutes and was done in a laminar flow cabinet for sterile work.

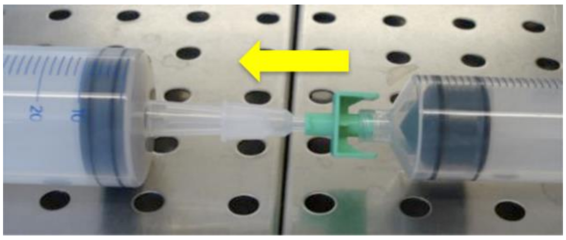

Figure S1. Description of the dispensing connector used for the transfer of the liposuction.

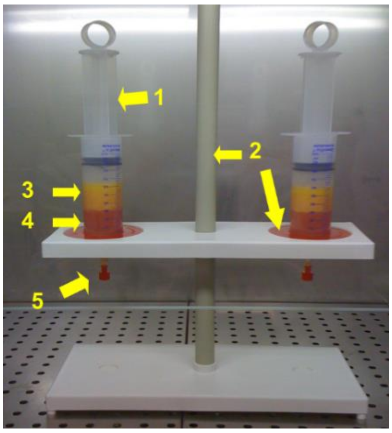

Figure S2. Description of the equipment used to extract the SVF from 50 mL (~ 50 g) human adipose tissue. A 100 mL syringe (1) was used as a separatory funnel to extract the cells of the SVF from fat. It was held in a vertical position in the laminar flow cabinet for sterile work by a laboratory support stand with support rings (2). Upper phase containing the adipose tissue (3). Lower layer containing the cells (4). Universal Closing Stopper attached to Luer Adaptor (5).

To dissociate the homogenized or suctioned fat, 10 mL DPBS (with Ca^{2+}, Mg^{2+}), containing the appropriate amount of Collagenase (# LS004147, Type B, Animal Origin Free, Worthington, Biochemical Corp., Lakewood, NJ) to reach the final concentration of 0.28 Wünsch U/mL enzyme, was added by pulling the piston of the syringe. In liquid aspiration procedures, it is useful to use a needle (e.g., # 4665473, 100 Sterican 14G x 31/8", BBraun, Melsungen, Germany) or, to make the process safe and avoid punctures, one can use a plastic cannula, for example, "Einmalknopfkanüle Steril DM 2.0/1.0 L:100 mm" (# 8572799, Polymed Medical Center, Glattbrugg, Switzerland). After 45 min incubation at 37^{0}C under constant but gentle agitation, 30 to 40 mL of a DPBS (without Ca2+, Mg2+, # L0615-500, Biowest, France) solution with 1% injectable human albumin (# 22918180119611, CSL Behring AG, Bern, Switzerland) were aspirated. The syringe was thoroughly shaken to extract the cells. The syringe was then returned to the support stand ring to allow for the separation of the two phases. The lower layer, which contained the SVF, was carefully pushed out into a conical 50 mL centrifuge tube (TPP, Trasadingen, Switzerland, # 91050). The extraction step can be repeated with 30 mL DPBS/1% injectable human albumin solution. Finally, after the sequential filtration, first through a 100 μm and then a 40 μm sieve (Cell Strainer, BD Falcon, Basel, Switzerland, # 352360 & # 352340), the SVF was centrifuged (600 g, 5 min). The pellet was resuspended in DPBS (without Ca2+, Mg2+)/1% injectable human albumin or in the tissue culture medium. The steps which followed the digestions with the Collagenase are schematically depicted in Figure S3. Finally, we believe the whole protocol could be easily translated into a GMP-compliant version to produce hASCs for cell therapeutic applications. The utilization of this procedure for commercial purposes is covered by the patent EP2726602B1 [1]

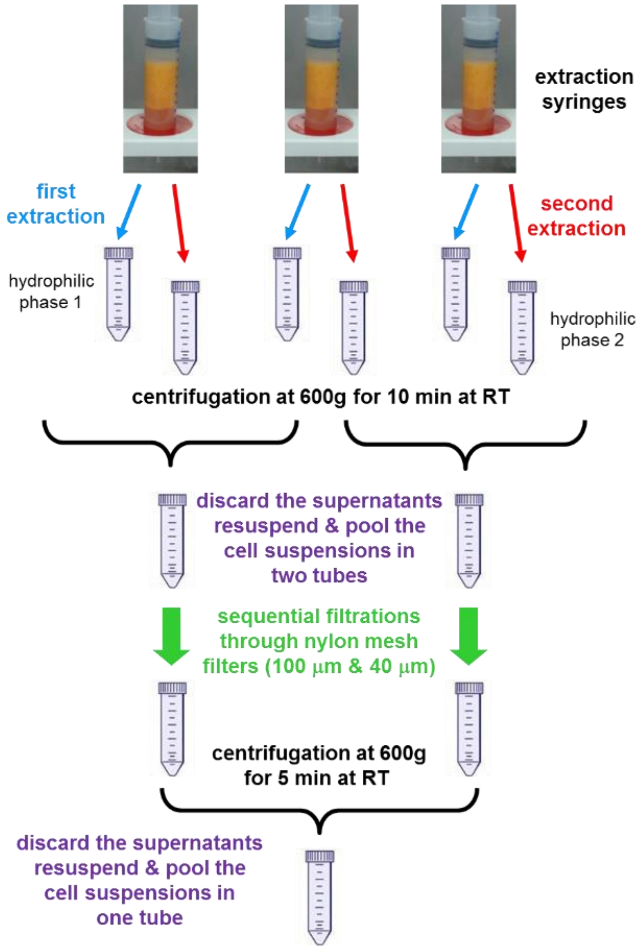

Figure S3. Diagram depicting the steps following the digestion with the Collagenase.

S2. Characterization of the cells of the SVF from adipose tissue

The SVF is a heterogeneous mixture of cells isolated by enzymatic dissociation of the adipose tissue. Adipocytes represent roughly two-thirds of the total cell extracted, and the rest are blood-derived cells, vascular cells, endothelial cells, smooth muscle cells, pericytes, fibroblasts, and ASCs. We developed a very informative multiparameter flow cytometry assay to characterize the cells of the SVF. This test allows us to subdivide the CD45-negative cells of the SVF into four fractions/subpopulations (see Figure 1).

This analysis provides the information necessary for the basic operations with these cells, like for example, the determination of the absolute cell number for every subtype (cells/mL)

- Crucial for seeding the cells at the optimal density for culture in serum-free conditions
- Crucial if one wants to cryopreserve the SVF for later use
- Crucial if the cells of the SVF are immediately used after the extraction (e.g., aesthetic surgery treatments).

S3. Initial plating of cells of the SVF for expansion

In serum-free conditions, the initial plating density is often the critical factor that determines whether a primary hASC-culture succeeds in taking root or not. Therefore, we elaborated the following rules to culture SVF cells in *UrSuppe* medium successfully: With manually Fibronectin-coated or commercially available vessels like Corning PureCoatTM ECM Mimetic Fibronectin Peptide, we usually plate:

- At least 3 x 10^5 nucleated cells/cm^2 in *UrSuppe* medium

- At least 3 x 10[4] ASCs/cm[2] in *UrSuppe* medium

Knowing the exact number of hASCs (defined as CD45-, CD146-, CD36-, CD34+, and CD73+ cells) contained in the SVF enormously facilitates the growth of these cells in SF conditions and the gain of a first confluent hASCs-culture at passage 0 (P0). If it is impossible to perform an accurate flow cytometric analysis to determine the number of hASCs present in the SVF, then at least 300,000 nucleated cells per cm^2 should be seeded. As shown in Figure S4, we found, on average, the hASCs represent 10% of the cells that compose the SVF. Therefore, with at least 300,000 nucleated cells of the SVF plated, there is the chance to hit the optimal minimal seeding density, which allows for the establishment of a hASCs-culture at P0 in SF conditions. Anyway, when the exact number of hASCs present in the SVF is not known, it is wise to seed 2-3 different cell densities.

After 3 to 5 days of culture, we gently rock the vessels to suspend and aspirate most non-adherent floating cells. After adding fresh *UrSuppe* medium (we do not recommend washing the cells at this stage), we continue culturing until confluency is reached. From now on, only 50% of the old medium should be replaced with fresh medium. Refer to Figure S5 for images of cultured ASCs.

After extraction of the SVF from human adipose tissue, we do not lyse the erythrocytes present in the cell suspension. This strategy is because we try to avoid further stressing the cells that were subjected to a harsh extraction protocol of at least two hours in the presence of proteases. However, in some preparations, there are indeed too many erythrocytes. In these cases, after two or three days of SVF culture, we gently collect the floating cells and lyse the erythrocytes using standard reagents and protocols. The RBC-free cells can be put back into the starting vessels or seeded in new ones. Therefore, this simple procedure has often augmented the yield of P0 hASCs. Finally, we realized that eliminating erythrocytes is certainly superfluous when the SVF, immediately after extraction, is meant to be cryopreserved. Indeed, when the samples are thawed, all erythrocytes are automatically lysed.

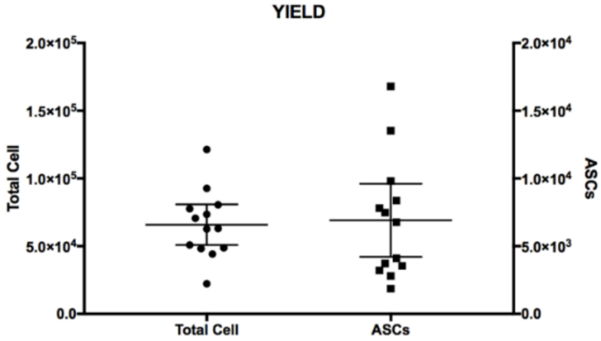

Figure S4. Boxplot representing mean and 95% confidence interval about the number of total cells and ASCs. ASCs scale is on the right and shows that they represent 10% of the total nucleated cells. The yield on the Y-axis indicates the number of cells for each gram of tissue. Data were analyzed with Graph Pad Prism software, n=13.

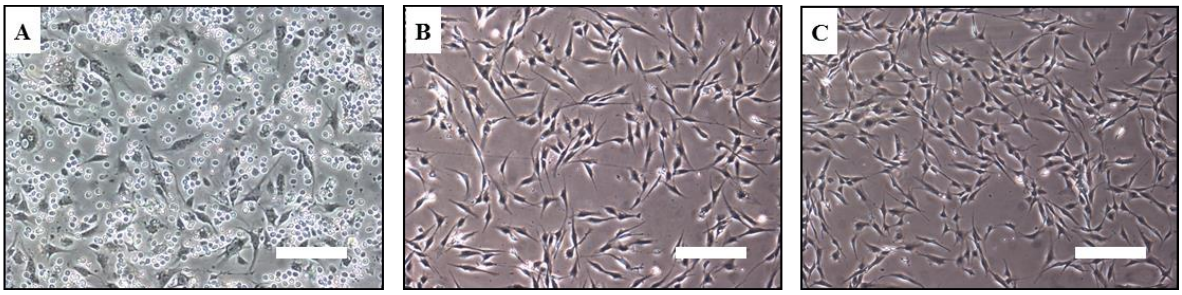

Figure S5. ASCs were grown in SF conditions. **(A)** Adherent cells of the SVF 24 hours after the seeding. Passage 1 **(B)** and passage 2 **(C)** of human ASCs. The cells display the typical spindle-shaped morphology of ASCs. Fold magnification 40X. Scale bar 250 μm.

S4. Basic guidelines for working with serum-free (SF) media

The challenge for every serum-free medium is to allow the growth of a primary culture (passage 0, P0) of ASCs. Indeed, after reaching the first confluency, the culture is relatively easy to passage, also in serum-free conditions. From this point on, the strategies for amplifying the cells are similar to those used with FBS-based media. After having placed dozens of different fresh or thawed SVF samples in culture with our serum-free *UrSuppe* medium, we have identified the critical points which determine whether the primary culture can take root or not. These are:

- Human lipoaspirate tissue samples must be processed up to 24 h after surgery
- Before processing, store the lipoaspirates at room temperature (not at 4 °C)
- Lipoaspirates must be sterile and avoid contamination with common and widespread commensal skin microorganisms
- Use commercially available coated vessels: Excellent with Corning PureCoat ECM Mimetic Fibronectin Peptide. Not always good with manually coated vessels
- Seeding density: See below, "Initial plating of cells of the SVF for expansion."
- Medium changes: At the beginning, not too often and replace only 50% of the old medium with fresh medium (for a possible explanation of this strategy, please see below)
- Excellent quality reagents (e.g., LPS-free) and components of the medium should be used, as well as very good quality instrumentation (mostly the incubator)
- Skill and training of the operator

Taken together, when the points listed above are respected, the success rate with our serum- and xeno-free cell *UrSuppe* culture medium is very high.

In SF conditions, cells secrete many paracrine factors that complement the synthetic medium and contribute to the cells' survival and growth. Therefore, it is crucial, especially with stem cells' primary cultures, not to completely replace the old medium. Therefore, it is wise to keep 25 to 50% of the old, "conditioned" medium. If any dead cells and debris are present, transfer the old medium to a 15 or 50 mL tube and centrifuge it for a few minutes (600 g) to remove the dead cells. The supernatant is then filtered through a 0.45 μm filter to remove the small debris. When the cells density is low with primary cultures, do not change the medium too often, the first time after 4 to 5 days of culture. It is wise to replace 80% of the old medium at high cell density every third day.

Serum-free media usually do not contain protease inhibitors. Therefore, it is necessary to wash the cells at least once to remove the protease before reseeding them in SF medium. Without this step, the cells could be irreversibly damaged by the presence of the proteolytic enzymes used to detach them from the culture vessel. The serum contains attachment and spreading factors, so it is usually unnecessary to coat the culture vessels when working with serum-containing media. On the other hand, working in SF conditions involves the use of extracellular matrix (ECM) coated tissue culture plasticware components. We recommend using Fibronectin (FN) since it has been shown that this ECM protein contributes, together with PREF-1, to "sealing" the stemness of the hASCs.

S5. Cryopreservation and thawing of the SVF

Developing effective techniques for the cryopreservation of hASCs could increase the usefulness of these cells in tissue engineering and regenerative medicine. Consistently with what has been done previously with the development of a serum-free medium to culture ASCs, we developed a serum-free cryopreservation medium, and we defined a controlled cooling rate protocol (Figure S6) for freezing and storing the SVF or ASCs in liquid nitrogen. This is the best procedure for the cryopreservation of cells. However, this procedure is feasible only if one has a controlled rate freezing device.

Our simple but effective cryopreservation medium is composed of:

- MEM Alpha w/o Phenol Red (# 1-23F22-I, BioConcept, Switzerland)

- 10% Cryosure Dex 40 (55% w/v DMSO USP Grade, 5% w/v Dextran 40 USP Grade, WAK - Chemie Medical GmbH; Germany)
- 1% injectable human albumin (# 22918180119611, CSL Behring AG, Switzerland)

If you do not have a freezing device available or for practical reasons to simplify your work, you can, of course, freeze down efficiently the cells with traditional systems, for example, using «Mr. Frosty Cryo 1 °C Freezing Container» (Nalgene, Thermo Fisher Scientific, USA) with the freezing medium "Synth-a-Freeze CTS" (Thermo Fisher Scientific, Waltham, USA). A suitable serum-free cryopreservation medium which could be prepared in the lab has been described by Yanela González Hernández and René Fischer. FILOCETH is composed of:

- 89% medium (TurboDoma, Cell Culture Technologies, Switzerland)
- 10% DMSO
- 1% Pluronic F68™

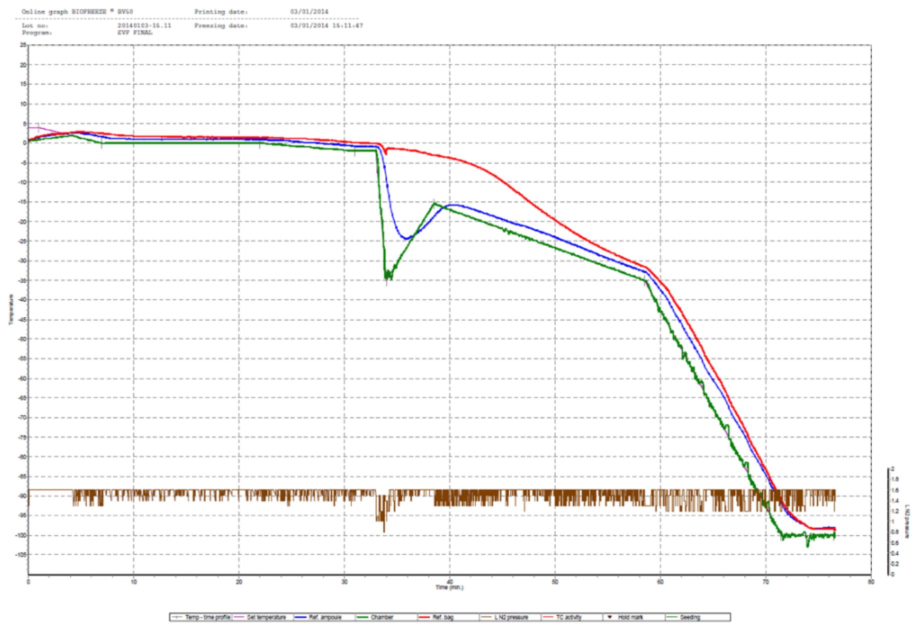

Figure S6. Controlled cooling rate protocol for the cryopreservation of freshly extracted cells of the SVF. The graph shows the final protocol to freeze the SVF extracted from adipose tissue. The red line represents the sample's temperature in the freezing medium, the blue one is water, and the green one is the chamber. The fusion point for our freezing medium, which contains 5.5% of DMSO, is around -3 °C. With this controlled cooling rate protocol, the temperature rises because the latent heat is contrasted by a quick temperature fall, so the released heat does not exceed the temperature of -3 °C (the fusion point of the cell suspension), avoiding in this way thawing of the sample.

The thawing protocol consists of a dilution system. Slow thaw the vial of cells by dilution with complete *UrSuppe* medium and subsequently seed the cells without washing or centrifuging. After 5-6 hours or the next day, when the cells have adhered, replace the entire medium.

S6. Evaluation of growth-related parameters

Based on the regular measurements of cell density and substrate/metabolite concentration, growth-dependent parameters were calculated for the planar cultivations as follows:

(I) Specific growth rate μ (Eq. 1):

$$\mu = \frac{ln\left(X_A(t)\right) - ln\left(X_A(0)\right)}{\Delta t} \quad (1)$$

Where μ is the net specific growth rate. $X_A(t)$ and $X_A(0)$ are the cell numbers at the end and the beginning of the exponential growth phase, respectively, and t is the time.

(II) Doubling time t_d (Eq. 2):

$$t_d = \frac{ln(2)}{\mu} \tag{2}$$

Where td is the doubling time, ln(2) the binary logarithm of 2, and μ the specific growth rate.

(III) Population Doubling Level PDL (Eq. 3):

$$PDL = \frac{1}{log(2)} \cdot log\left(\frac{X_A(t)}{X_A(0)}\right) \tag{3}$$

Where PDL is the number of population doublings, and $X_A(0)$ and $X_A(t)$ are the cell numbers at the beginning and the end of the cultivation, respectively.

(IV) Expansion factor EF (Eq. 4):

$$EF = \frac{X_A(t_{max})}{X_A(t=0)} \tag{4}$$

Where EF is the expansion factor, $X_A(_{tmax})$ is the maximum cell number, and $X_A(_{t=0})$ is the inoculated cell number.

(V) Lactate yield from glucose $Y_{Lac/Glc}$ (Eq. 5):

$$Y_{Lac/Glc} = \frac{\Delta Lac}{\Delta Glc} \tag{5}$$

Where $Y_{Lac/Glc}$ is the lactate yield from glucose, ΔLac is the lactate production over a specific time period. ΔGlc is the glucose consumption over the same time period (= exponential growth phase).

(IV) Specific metabolite flux q_{met} (Eq. 6):

$$q_{met} = \left(\frac{\mu}{X_A(t)}\right)\left(\frac{C_{met}(t) - C_{met}(0)}{e^{\mu t} - 1}\right) \tag{6}$$

Where q_{met} is the net specific metabolite consumption or production rate (for Glc, Lac, Amn), μ is the specific cell growth rate, XA(t) is the cell number at the end of the exponential growth phase, $C_{met}(t)$ and $C_{met}(0)$ are the metabolite concentrations at the end and the beginning of the exponential growth phase, respectively, and t is the time.

Table S2. Detailed information of the antibodies used for the flow cytometric measurements

Name	# Catalog	Company	Amount for SVF [ng]	Amount for ASC [ng]
7-AAD	559925	Becton Dickinson	2.5 µL	2.5 µL
CD26-FITC	302704	BioLegend	-	50
CD34-BV650	343624	BioLegend	125	-
CD34-FITC	130-113-178	Miltenyi	-	50
CD36-APC	130-095-475	Miltenyi	55	50
CD36-FITC	130-120-064	Miltenyi	-	50
CD45-PC7	304016	BioLegend	125	-
CD54-PE	12-0549-42	Thermo Fisher	-	50
CD55-BV421	742677	Becton Dickinson	-	50
CD73-FITC	344016	BioLegend	75	50
CD90-APC	328113	BioLegend	-	50
CD95-FITC	305605	BioLegend	-	50
CD105-PE	323206	BioLegend	-	50
CD106-PC7	25-1061-80	Thermo Fisher	-	50
CD140a-APC	A15718	Thermo Fisher	-	50
CD142-APC	365206	BioLegend	-	50

CD146-PE	130-092-853	Miltenyi	34	50
CD248-BV421	743899	Becton Dickinson	-	50
SYTO™40	S11351	Thermo Fisher	5 μM	-

Table S3. Reverse transcription detailed procedure

Reagent	Amount
Mix 1:	
RNA	Up to 5 μg
Oligo dT	0.5 μg
Random Primers	0.5 μg
H_2O	Final volume 5 μL
Mix 2:	
Buffer 5x	2 μL
$MgCl_2$	1 μL [2.5 mM]
dNTPs	0.5 μL [0.5 mM]
Inhibitor RNase	0.25 μL [20 Units]
RT Enzyme	0.5 μL
H_2O	Final volume 5 μL
***Procedure*:**	
Add Mix 1, incubate 5 min at 70 °C, cool to 10 °C and incubate 5 min in ice	
Add Mix 2 and incubate 5 min at 25 °C, 42 °C for 1h, and 70 °C for 15 min	

Table S4. Primers sequences

Gene Name	Forward Primer (5′-3')	Reverse Primer (5′-3')
ACTB	CTG GAA CGG TGA AGG TGA CA	AAG GGA CTT CCT GTA ACA ATG CA
ADIPONECTIN	AGG CCG TGA TGG CAG AGA TG	ACT CCG GTT TCA CCG ATG TC
*CD26**	AGT GGC ACG GCA ACA CAT T	AGA GCT TCT ATC CCG ATG ACT T
CD34	TGG CTG TCT TGG GCA TCA CTG G	CTG AAT GGC CGT TTC TGG AGG TGG
*CD36**	TGT GCA AAA TCC ACA GGA AGT G	CCT CAG CGT CCT GGG TTA CA
*CD55**	AGA GTT CTG CAA TCG TAG CTG C	CAC AAC AGT ACC GAC TGG AAA AT
*CD142**	GGC GCT TCA GGC ACT ACA A	TTG ATT GAC GGG TTT GGG TTC
*CD146**	AGC TCC GCG TCT ACA AAG C	CTA CAC AGG TAG CGA CCT CC
*CD248**	AGT GTT ATT GTA GCG AGG GAC A	CCT CTG GGA AGC TCG GTC TA
*DKK1**	ATA GCA CCT TGG ATG GGT ATT CC	CTG ATG ACC GGA GAC AAA CAG
DLL1	ACT CCG CGT TCA GCA ACC CCA T	TGG GTT TTC TGT TGC GAG GTC ATC AGG
FABP4 [2]	CAG TGT GAA TGG GGA TGT GA	CGT GGA AGT GAC GCC TTT
KLOTHO-α	GCT CTC AAA GCC CAC ATA CTG	GCA GCA TAA CGA TAG AGG CC
KLOTHO-β	AGA AGA CAC CAC GGC CAT CTA C	CAT TCA AAG CCA TCC AGG AGA G
*LEPTIN**	TGC CTT CCA GAA ACG TGA TCC	CTC TGT GGA GTA GCC TGA AGC
*NOTCH1**	TGG ACC AGA TTG GGG AGT TC	GCA CAC TCG TCT GTG TTG AC
PGC1α	GCT TTC TGG GTG GAC TCA AGT	GAG GGC AAT CCG TCT TCA TCC

PPARG[3]	TGA CAG CGA CTT GGC AAT ATT TAT T	TTG TAG CAG GTT GTC TTG AAT GTC T
PREF1[2]	TGA CCA GTG CGT GAC CTC T	GGC AGT CCT TTC CCG AGT A
RUNX2 *	TCA ACG ATC TGA GAT TTG TGG G	GGG GAG GAT TTG TGA AGA CGG
SOX9 *	AGC GAA CGC ACA TCA AGA C	CTG TAG GCG ATC TGT TGG GG
*UCP1**	AGG ATC GGC CTC TAC GAC AC	GCC CAA TGA ATA CTG CCA CTC
WISP1	CGA GGT ACG CAA TAG GAG TGT G	GAA GGA CTG GCC GTT GTT GTA G
*WISP2**	GCG ACC AAC TCC ACG TCT G	TCC CCT TCC CGA TAC AGG C
ZFP423[3]	GAT CAC TGT CAG CAG GAC TT	TGC CTC TTC AAG TAG CTC A
ZFP521[3]	GGC TGT TCA AAC ACA AGC G	GCA CAT TTA TAT GGC TTG TTG

* Primer from PrimerBank (https://pga.mgh.harvard.edu/primerbank/)

Table S5. RT-qPCR cycle conditions

**Phase**	**T (°C)**	**Time (min)**	**Repetition**
Denaturation	95 °C	2:00	
Denaturation	95 °C	0:05	} X40
Annealing + Extension	60 °C	0:20	} X40
Denaturation	95 °C	0:05	
Melting Curve	65-95 °C	18:00	

Supplementary Information for the Results Section

Table S6, S7, and S8 provide an overview and short description of genes measured in this study.

Table S6. Overview of measured *Stemness Maintenance Genes*

Name	Description	Reference
PREF1 (DLK1)	Preadipocyte Factor 1 (Delta-Like 1 homolog) is a transmembrane protein that inhibits adipogenesis, and it belongs to the non-canonical Notch ligands family (together with Dlk2). Pref1 also exists as a biologically active soluble form, but its receptor is still unknown.	Hudak *et al.* [4]<br>Hei *et al.* [5]
SOX9	Sox9 is a member of the HMG-box class DNA-binding proteins and is a Pref1 target. It directly binds to c/EBPb and c/EBPd promoters to suppress their promoter activity, thus repressing adipocyte differentiation.	Wang *et al.* [6]
ZFP521	Zinc Finger Protein 521 is a transcription factor that inhibits adipogenesis. ZFP521 binds to EBF1 and inhibits its transcriptional activity. This leads to a substantially attenuated expression of the proadipogenic factor ZFP423.	Kang *et al.*[3]<br>Chiarella *et al.* [7]
WISP2	Wnt1-inducible signaling pathway protein 2 is an endogenous and secreted auto/paracrine non-conventional WNT ligand, promoting the proliferation of precursors cells and inhibiting their adipogenic commitment and differentiation.	Grünberg *et al.* [8]<br>Hammarstedt *et al.* [9]<br>Grünberg *et al.* [10]
NOTCH1	It regulates the proliferation and differentiation of the adipocyte progenitors' cells. Notch1 inhibits differentiation through its target gene, HesI, which can directly repress the expression of c/EBPa and PPARG. HesI decreases during adipogenesis.	Shan *et al.* [11]<br>Ross *et al.* [12]
DLL1	Delta-like protein 1 is one of the five canonical Notch ligands. It inhibits adipogenesis.	Murata *et al.* [13]<br>Sparling *et al.* [14]

Table S7. Overview of measured *Differentiation Regulators/Markers*

Name	Description	Reference
PPARG	Peroxisome Proliferator-Activated Receptor gamma is a ligand-dependent transcription factor that is a member of the nuclear hormone receptor superfamily. It plays a crucial role in adipose tissue development and differentiation.	Ahmadian *et al.* [15]<br>Barak *et al.* [16]<br>Rosen *et al.* [17]<br>Tontonoz *et al.* [18]
ZFP423	Zinc Finger Protein 423 is responsible for adipogenic commitment. It induces PPARG expression and terminal adipogenic differentiation.	Gupta *et al.* [19]<br>Gupta *et al.* [20]
RUNX2	Runx2 is a transcription factor that is essential for osteoblast differentiation and chondrocyte maturation.	Toshihisa *et al.* [21]<br>Komori *et al.* [22]
WISP1	The Wnt1-inducible signaling pathway protein-1 increases during adipocyte differentiation, thus stimulating adipogenesis in humans. It is both an intracellular and a secreted protein found in the extracellular matrix. In mice, the effect of WISP1 on adipogenesis was opposite to what has been reported in humans.	Ferrand *et al.* [23]<br>Murahovschi *et al.* [24]

DKK1	Dickkopf1 inhibits the Wnt signaling and promotes differentiation. It binds as high-affinity antagonists to LRP5/6 co-receptors, thus preventing Wnt-ligand induced receptor complex formation.	Christodoulide *et al.* [25]<br>Gustafson *et al.* [26]
CD34	CD34 is a transmembrane phosphoglycoprotein expressed on precursors cells and mature adipocytes. Its function on the adipocyte membrane remains to be determined.	Festy *et al.* [27]<br>Sidney *et al.* [28]<br>Scherberich *et al.* [29]
CD36	CD36 is a transmembrane glycoprotein classified as a class B scavenger receptor. It imports fatty acids inside cells and plays a functional role in adipocyte differentiation and adipogenesis.	Christiaens *et al.* [30]<br>Gao *et al.* [31]
CD146	Three forms of this adhesion protein have been described, including two transmembrane isoforms and a soluble protein, detectable in the plasma. Its expression increases during adipogenic differentiation.	Leroyer *et al.* [32]<br>Walmsley *et al.* [33]
α-KLOTHO	α-Klotho promotes adipogenesis through CCAAT/enhancer-binding proteins (cEBPs) and PPARG, which collectively maintain adipocyte gene expression to terminate preadipocyte differentiation.	Fan *et al.* [34]<br>Zhang *et al.* [35]<br>Razzaque *et al.* [36]
β-KLOTHO	β-klotho, which is highly conserved and localized to the cell membrane, is expressed predominantly in the liver and white adipose tissue.	Ito *et al.* [37]
FABP4	Fatty acid-binding protein 4, also called aP2 (adipocyte protein 2), is a carrier protein for fatty acids primarily expressed in adipocytes.	Burls *et al.* [38]<br>Harms *et al.* [39]
ADIPONECTIN	Adiponectin is a protein hormone that modulates several metabolic processes and is secreted from adipose tissue.	Lee *et al.* [40]<br>Stern *et al.* [41]<br>Villarroya *et al.* [42]
LEPTIN	Leptin is a hormone predominantly made by mature adipose cells regulating energy balance by inhibiting hunger and diminishing fat storage in adipocytes.	Harms *et al.* [39]<br>Stern *et al.* [41]<br>Caputo *et al.* [43]
UCP1	Thermogenin is an uncoupling protein found in the mitochondria of brown adipose tissue (BAT). It is used to generate heat by non-shivering thermogenesis.	Villarroya *et al.* [42]<br>Tamucci *et al.* [44]<br>Wang *et al.* [45]
PGC1α	PGC1α is the master regulator of mitochondrial biogenesis, and it is responsible for β-aminobutyric acid secretion. The effect of β-aminobutyric acid in white fat includes the activation of thermogenic genes that prompt white adipose tissue browning.	Villarroya *et al.* [42]<br>Nascimento *et al.* [46]

Table S8. Overview of measured *Lineage Hierarchy Markers*

Name	Description	Reference
CD26	Dipeptidyl peptidase-4 (DPP4), also known as adenosine deaminase complexing protein 2 or CD26, is an enzyme expressed on the surface of most cell types and is associated with immune regulation, signal transduction, and apoptosis. It is a type II transmembrane glycoprotein, but a soluble form is present in blood plasma and various body fluids. A large number of human chemokines, including CXCL12/SDF-1, are cleaved by CD26, and this has prominent effects on their receptor binding, signaling, and hence, in vitro and in vivo biologic activities	Mortier *et al.* [47]<br>Metzemaekers *et al.* [48]<br>Merrick *et al.* [49]<br>Rennert *et al.* [50]

CD55	Complement decay-accelerating factor, also known as CD55 or DAF, is a protein that, in humans, regulates the complement system on the cell surface.	Merrick *et al*. [49]<br>Rennert *et al*. [50]
CD142	Tissue factor, also called platelet tissue factor, factor III, or CD142, is a protein present in subendothelial tissue and leukocytes. It is the primary initiator of the blood coagulation cascade, which ensures rapid hemostasis in the case of organ damage. It also has signaling activity and promotes inflammatory responses via protease-activated receptors in concert with other coagulation factors.	Schwalie *et al*. [51]<br>Chu *et al*. [52]
CD248	CD248, also known as endosialin and tumor endothelial marker 1 (TEM-1), plays a role in cell-cell adhesion processes and host defense. This marker does not have a fully characterized role, but its expression has been associated with angiogenesis in the embryo and uterus and tumor development and growth. CD248 could characterize pro-angiogenic subpopulation of SVF cells.	Merrick *et al*. [49]<br>Brett *et al*. [53]

S7. Differentiation assay to verify hASC multipotency

To confirm that cells isolated from adipose tissue and cultured in *UrSuppe* maintain their multipotency, we perform a differentiation assay. Cells were expanded until passage 2 (P2), changing the medium every 2 days and splitting them at around 80 % of confluence. After reaching P2 the cells were used for multilineage differentiation assay using a commercial induction medium.

Briefly:

- hMSC differentiation BulletKit™ adipogenic (Lonza, Basel, Switzerland #PT-3004) were used for adipogenic assay: cells were seeded at 20’000 cells/cm^2 in a 6 wells multiwell in basal medium, after 24 hours induction was started with induction medium. The medium was changed every 7 days, induction continue till day 19.
- hMSC differentiation BulletKit™ osteogenic (Lonza, Basel, Switzerland #PT-3002) was used for the osteogenic assay. The cells were seeded at 3’000 cells/cm^2 in a 6 wells multiwell in basal medium. After 24 hours induction was started with an induction medium. The medium was changed every 3-4 days, induction continue till day 19
- hMSC differentiation BulletKit™ chondrogenic (Lonza, Basel, Switzerland #PT-3003) was used for chondrogenic assay: cells were pelleted at 250’000 cells/pellet in different 15 mL TPP tubes in differentiation BulletKit medium. The medium was changed every 3-4 days, induction continue till day 19.

All manipulations were done according to the manufacturer's instructions and recommendations. After 19 days of induction, we performed histochemical assays with the samples. For the adipogenic and osteogenic induced samples: Cells were fixed with a 4% formaldehyde/0.2% glutaraldehyde solution for 20 minutes and washed three times with PBS. After that, the cells were incubated with the specific staining solution: Oil-Red-O (Sigma-Aldrich, cat no. 75087) to detect fat vacuoles in the cells (adipo-induction) and Alizarin-Red S (Sigma-Aldrich, cat. no. 05600) to stain calcium-rich extracellular matrix (osteo-induction). For the chondrogenic induced samples: The high-density micromass pellets were fixed in 4% paraformaldehyde, embedded in paraffin, and cut into 5μm thick sections with a rotation microtome to examine chondrogenesis. The micro-slices were put onto adhesive microscopic slides, dried on a heating plate for half an hour, and dried overnight at 40°C. Afterward, the microscopic slides were stained with Alcian Blue (Sigma-Aldrich, cat. no. A3157) to highlight the regions saturated with an extracellular matrix composed of acidic polysaccharides that are usually highly expressed in cartilage (chondro-induction). After the stainings, all samples were documented and analyzed, and Figure S7 shows the results of a

representative trilineage differentiation assay. These tests confirmed that hASCs expanded with the *UrSuppe* medium remained undifferentiated and capable of trilineage differentiation.

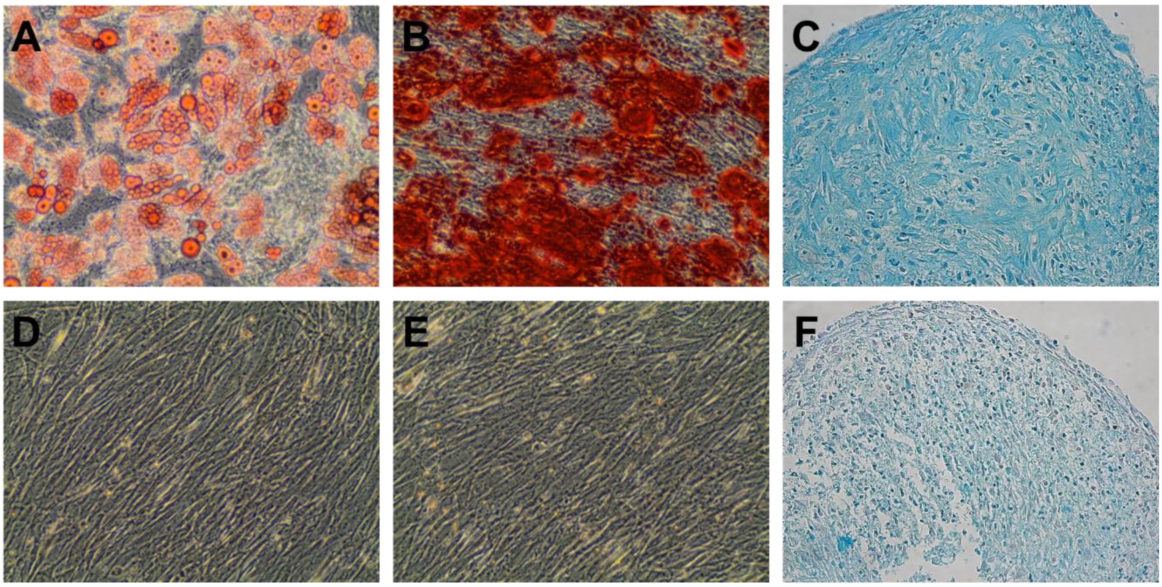

Figure S7. Multipotency assay with hASCs grown in *UrSuppe* medium. Representative images of a trilineage differentiation assay of hASCs obtained from one donor. **(A)** Oil-Red-O coloration to highlight fat vesicles after 19 days of induction. No lipids droplets were detected in the control sample (**D**). Magnification 100x. **(B)** Alizarin-Red S coloration to stain calcium-rich extracellular matrix regions specific of osteocytes after 19 days of induction. No significant calcium-deposits were found in the control sample (**E**). Magnification 100x. **(C)** Slice from micromass cell pellet-induced culture to highlight the extracellular matrix composed of acidic polysaccharides that are usually found in the cartilage. The slice was stained with Alcian-Blue after 19 days of induction. Low-intensity staining of the control micromass cell pellet cultured in *UrSuppe* medium (**F**). Magnification: 40x.

S8. Proteome analysis

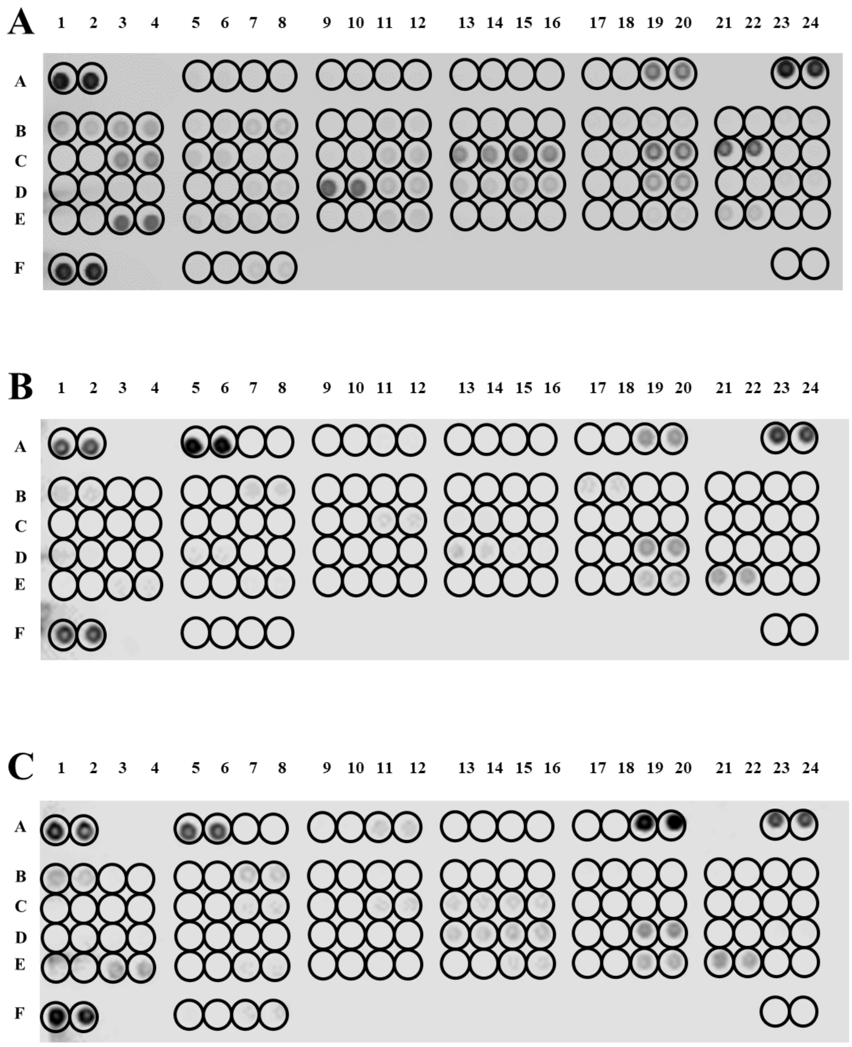

Figure S8. Comparing the secretion profile of hASCs. **(A)**: Profile of hASCs cultured in the classical static 2D cell culture system in *UrSuppe*. **(B)**: Profile of hASCs differentiated in WAT. **(C)**: Profile of hASCs differentiated in BAT. Array processing techniques according to the manufacturer of the kit (bio-techne, #ARY024).

Table S9. Qualitative evaluation of the factors secreted by undifferentiated hASCs and WAT or BAT induced cells. The qualitative evaluation for each detected factor is indicated by the number of X. Shallow expression is indicated by (X), whereas no expression by (-). Quantitative results are shown in Figure 11.

Group	Secreted Factor	Undifferentiated hASCs	WAT Induction	BAT Induction
1	Adiponectin	(X)	XXX	XX
	Leptin	-	X	-
2	Cathepsin D	XX	XX	XXX
	Cathepsin L	X	X	X
	Cathepsin S	X	-	-
3	Fetuin-B	-	X	-
	PAI-I/Serpin E1	(X)	X	X
	Timp-1	X	XX	XX
4	IL-6	XX	-	-
	IL-8/CXCL8	XX	-	-
	CCL2/MCP-1	X	-	-
	CCL5/Rantes	X	-	-
	M-CSF	X	X	X
	MIF	X	-	X
	Complement Factor D	X	X	X
	Pentraxin-3/TSG-14	XXX	X	XX
	Lipocalin-2	XX	-	-
5	IGFBP-4	X	X	X
	IGFBP-6	XX	X	X
	IGFBP-7	XX	-	X
6	HGF	XX	-	-
	VEGF	X	-	-
	Angiopoietin-like	-	-	X
7	CD26	X	-	-
	CD54	X	-	-
	Pref-1	X	-	-
8	Nidogen-1/Entactin	XX	XX	XX

Group legend. 1: adipogenic signature cytokines; 2: proteases; 3: proteases inhibitors; 4: inflammatory and innate immunity-related cytokines and chemokynes; 5: anti-adipogenic factors; 6: angiogenic factors; 7: lineage hierarchy markers; 8: Protein of the extracellular matrix (ECM).

Table S10. Human adipokine array

Table/List on the left: Coordinates and explanations of the 58 different spots present on the commercial human adipokine array membrane.

Coordinate	Analyte/Control	Coordinate	Analyte/Control
A1, A2	Reference Spots	C19, C20	IL-6
A5, A6	Adiponectin/Acrp30	C21, C22	CXCL8/IL-8
A7, A8	Angiopoietin-1	C23, C24	IL-10
A9, A10	Angiopoietin-2	D1, D2	IL-11
A11, A12	Angiopoietin-like 2	D3, D4	LAP (TGF-β1)
A13, A14	Angiopoietin-like 3	D5, D6	Leptin
A15, A16	BAFF/BLyS/TNFSF13B	D7, D8	LIF
A17, A18	BMP-4	D9, D10	Lipocalin-2/NGAL
A19, A20	Cathepsin D	D11, D12	CCL2/MCP-1
A23, A24	Reference Spots	D13, D14	M-CSF
B1, B2	Cathepsin L	D15, D16	MIF
B3, B4	Cathepsin S	D17, D18	Myeloperoxidase
B5, B6	Chemerin	D19, D20	Nidogen-1/Entactin
B7, B8	Complement Factor D	D21, D22	Oncostatin M (OSM)
B9, B10	C-Reactive Protein/CRP	D23, D24	Pappalysin-1/PAPP-A
B11, B12	DPPIV/CD26	E1, E2	PBEF/Visfatin
B13, B14	Endocan	E3, E4	Pentraxin-3/SG-14
B15, B16	EN-RAGE	E5, E6	Pref-1/DLK-1/FA1
B17, B18	Fetuin B	E7, E8	Proprotein Convertase 9/PCSK9
B19, B20	FGF basic	E9, E10	RAGE
B21, B22	FGF-19	E11, E12	CCL5/RANTES
B23, B24	Fibrinogen	E13, E14	Resistin
C1, C2	Growth Hormone	E15, E16	Serpin A8/AGT
C3, C4	HGF	E17, E18	Serpin A12
C5, C6	ICAM-I/CD54	E19, E20	Serpin E1/PAI-1
C7, C8	IGFBP-2	E21, E22	TIMP-1
C9, C10	IGFBP-3	E23, E24	TIMP-3
C11, C12	IGFBP-4	F1, F2	Reference Spots
C13, C14	IGFBP-6	F5, F6	TNF-α
C15, C16	IGFBP-rp1/IGFBP-7	F7, F8	VEGF
C17, C18	IL-1β/IL-1F2	F23, F24	Negative Controls

S9. Other flow cytometry result

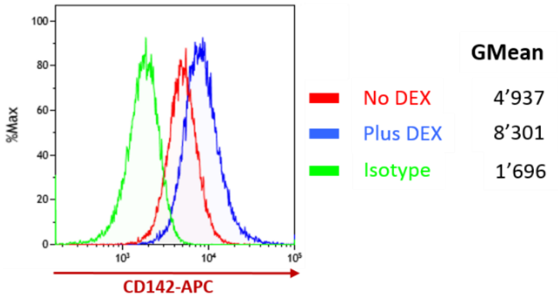

Figure S9. Representative flow cytometry expression of CD142 after Dexamethasone stimulation. Geometric mean expression of the cells cultured in *UrSuppe* with and without Dexamethasone (DEX). The graph shows the increased fluorescence intensity after stimulation with DEX. The above data are from a representative patient

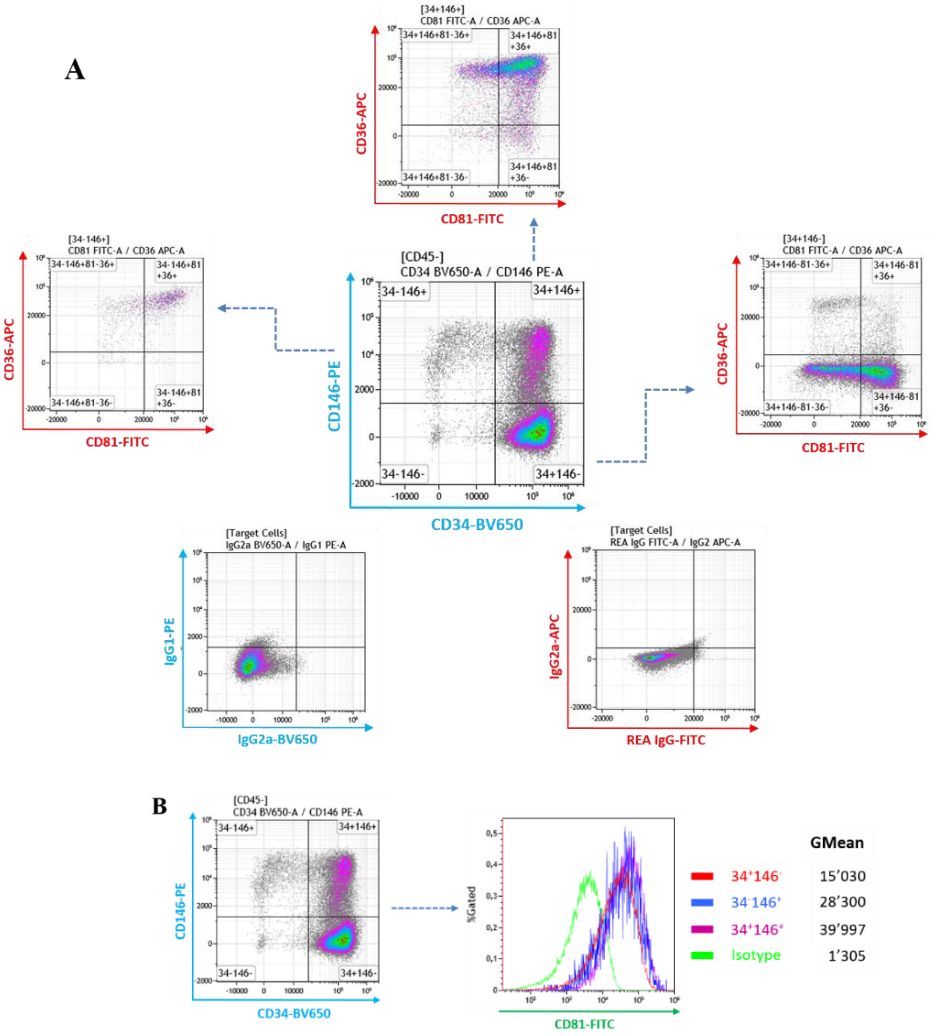

C

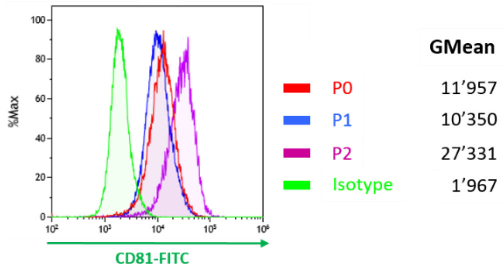

Figure S10. Representative flow cytometry distribution of CD81 in the cells of the SVF and during culture. **(A)** Analysis of the SVF cells extracted from human subcutaneous adipose tissue to check the CD81 expression. The same analysis strategy explained in Figure 1 from the body textwas used. Each of the three different subpopulations was analyzed to evaluate the co-expression of CD81 and CD36, which is a notorious marker signaling adipogenic differentiation **(B)** Geometric mean average of the CD81 expression in the different populations of the SVF. (**C)** Geometric mean expression of CD81 at three different passages (P0, P1, and P2) during culture in *UrSuppe* medium. The above data are from a representative patient.

S10. Different cell culture media used to culture hASCs

Table S11. List of different serum- and xeno-free media used to grow hASCs

Medium Name	Type	Coating Requirement	Direct Seeding after Isolation	Directly seeding after thawing	Reference
UrSuppe	Chemically Defined	Fibronectin	YES	YES	Jossen *et al*. [54]
MSC Nutristem Biological Industries[55][55]	Chemically Defined	Specific Attachment Substrate	YES	YES	Sagaradze *et al*. [55]
StemFit AMS-Bio	Chemically Defined	iMatrix-511	n/a	YES	
DXF Medium PromoCell	Chemically Defined	Fibronectin	n/a	YES	
STK1	Chemically Defined	None	YES	n/a	Lee *et al*. [56]
Invitrogen StemPro	Xeno and Serum-Free	CELL-Start Coating	YES	YES	Bakopoulou *et al*. [57]
MesenCult Stem Cell Technologies	Xeno and Serum-Free	Specific Attachment Substrate	YES	YES	Lensch *et al*. [58] Winnier *et al*. [59]
CNT-Prime MSC	Xeno and Serum-Free	None	YES	YES	
Xuri-MSC GE Healthcare	Xeno and Serum-Free	Xuri MSC Attachment solution	n/a	YES	Gerby *et al*. [60]
PowerStem MSC1 PanBiotech	Xeno-Free	Fibronectin	YES	YES	

Miltenyii Stem MACS	Xeno-Free	None	YES	YES	Bakopoulou *et al.* [57] Lensch *et al.* [58]
Stemline Sigma	Xeno-Free	None	n/a	n/a	

References

1. Soldati, G.; Tallone, T. High-safety process for the preparation of purified stem cell fraction. *EP2726602B1* 2012.
2. Mitterberger, M.C.; Lechner, S.; Mattesich, M.; Kaiser, A.; Probst, D.; Wenger, N.; Pierer, G.; Zwerschke, W. DLK1(PREF1) is a negative regulator of adipogenesis in CD105+/CD90+/CD34+/CD31-/FABP4- adipose-derived stromal cells from subcutaneous abdominal fat pats of adult women. *Stem Cell Res.* **2012**, *9*, 35–48, doi:10.1016/j.scr.2012.04.001.
3. Kang, S.; Akerblad, P.; Kiviranta, R.; Gupta, R.K.; Kajimura, S.; Griffin, M.J.; Min, J.; Baron, R.; Rosen, E.D. Regulation of Early Adipose Commitment by Zfp521. *PLoS Biol.* **2012**, *10*, e1001433, doi:10.1371/journal.pbio.1001433.
4. Hudak, C.S.; Gulyaeva, O.; Wang, Y.; Park, S. min; Lee, L.; Kang, C.; Sul, H.S. Pref-1 marks very early mesenchymal precursors required for adipose tissue development and expansion. *Cell Rep.* **2014**, *8*, 678–687, doi:10.1016/j.celrep.2014.06.060.
5. Hei, S.S. Minireview: Pref-1: Role in adipogenesis and mesenchymal cell fate. *Mol. Endocrinol.* **2009**, *23*, 1717–1725, doi:10.1210/me.2009-0160.
6. Wang, Y.; Sul, H.S. Pref-1 Regulates Mesenchymal Cell Commitment and Differentiation through Sox9. *Cell Metab.* **2009**, *9*, 287–302, doi:10.1016/j.cmet.2009.01.013.
7. Chiarella, E.; Aloisio, A.; Codispoti, B.; Nappo, G.; Scicchitano, S.; Lucchino, V.; Montalcini, Y.; Camarotti, A.; Galasso, O.; Greco, M.; et al. ZNF521 Has an Inhibitory Effect on the Adipogenic Differentiation of Human Adipose-Derived Mesenchymal Stem Cells. *Stem cell Rev. reports* **2018**, *14*, 901–914, doi:10.1007/s12015-018-9830-0.
8. Grünberg, J.R.; Hammarstedt, A.; Hedjazifar, S.; Smith, U. The novel secreted adipokine wnt1-inducible signaling pathway protein 2 (WISP2) is a mesenchymal cell activator of canonical WNT. *J. Biol. Chem.* **2014**, *289*, 6899–6907, doi:10.1074/jbc.M113.511964.
9. Hammarstedt, A.; Hedjazifar, S.; Jenndahl, L.; Gogg, S.; Grunberg, J.; Gustafson, B.; Klimcakova, E.; Stich, V.; Langin, D.; Laakso, M.; et al. WISP2 regulates preadipocyte commitment and PPARγ activation by BMP4. *Proc. Natl. Acad. Sci. U. S. A.* **2013**, *110*, 2563–2568, doi:10.1073/pnas.1211255110.
10. Grünberg, J.R.; Elvin, J.; Paul, A.; Hedjazifar, S.; Hammarstedt, A.; Smith, U. CCN5/WISP2 and metabolic diseases. *J. Cell Commun. Signal.* **2018**, *12*, 309–318, doi:10.1007/s12079-017-0437-z.
11. Shan, T.; Liu, J.; Wu, W.; Xu, Z.; Wang, Y. Roles of Notch Signaling in Adipocyte Progenitor Cells and Mature Adipocytes. *J. Cell. Physiol.* **2017**, *232*, 1258–1261, doi:10.1002/jcp.25697.
12. Ross, D.A.; Rao, P.K.; Kadesch, T. Dual Roles for the Notch Target Gene Hes-1 in the Differentiation of 3T3-L1 Preadipocytes. **2004**, *24*, 3505–3513, doi:10.1128/MCB.24.8.3505–3513.2004.
13. Murata, A.; Yoshino, M.; Hikosaka, M.; Okuyama, K.; Zhou, L.; Sakano, S.; Yagita, H.; Hayashi, S.I. An evolutionary-conserved function of mammalian notch family members as cell adhesion molecules. *PLoS One* **2014**, *9*, doi:10.1371/journal.pone.0108535.
14. Sparling, D.P.; Yu, J.; Kim, K.J.; Zhu, C.; Brachs, S.; Birkenfeld, A.L.; Pajvani, U.B. Adipocyte-specific blockade of gamma-secretase, but not inhibition of Notch activity, reduces adipose insulin sensitivity. *Mol. Metab.* **2016**, *5*, 113–121, doi:10.1016/j.molmet.2015.11.006.
15. Ahmadian, M.; Suh, J.M.; Hah, N.; Liddle, C.; Atkins, A.R.; Downes, M.; Evans, R.M. PPAR γ signaling and metabolism : the good, the bad and the future. *Nat. Med.* **2013**, *19*, 557–566, doi:10.1038/nm.3159.
16. Barak, Y.; Nelson, M.C.; Ong, E.S.; Jones, Y.Z.; Ruiz-Lozano, P.; Chien, K.R.; Koder, A.; Evans, R.M. PPARγ is required for placental, cardiac, and adipose tissue development. *Mol. Cell* **1999**, *4*, 585–595, doi:10.1016/S1097-2765(00)80209-9.

17. Rosen, E.D.; Sarraf, P.; Troy, A.E.; Bradwin, G.; Moore, K.; Milstone, D.S.; Spiegelman, B.M.; Mortensen, R.M. PPARγ is required for the differentiation of adipose tissue in vivo and in vitro. *Mol. Cell* **1999**, *4*, 611–617, doi:10.1016/S1097-2765(00)80211-7.
18. Tontonoz, P.; Hu, E.; Spiegelman, B.M. Stimulation of adipogenesis in fibroblasts by PPARγ2, a lipid-activated transcription factor. *Cell* **1994**, *79*, 1147–1156, doi:10.1016/0092-8674(94)90006-X.
19. Gupta, R.K.; Arany, Z.; Seale, P.; Mepani, R.J.; Ye, L.; Conroe, H.M.; Roby, Y.A.; Kulaga, H.; Reed, R.R.; Spiegelman, B.M. Transcriptional control of preadipocyte determination by Zfp423. *Nature* **2010**, *464*, 619–623, doi:10.1038/nature08816.
20. Gupta Rana K. et al. Zfp423 expression identifies committed preadipocytes and localizes to adipose endothelial and perivascular cells. *Cell Metab.* **2012**, *15*, 230–239, doi:10.1016/j.cmet.2012.01.010.
21. Toshihisa, K. Molecular Mechanism of Runx2-Dependent Bone Development. *Mol. Cells* **2020**, *43*, 168–175, doi:10.14348/molcells.2019.0244.
22. Komori, T. Runx2, an inducer of osteoblast and chondrocyte differentiation. *Histochem. Cell Biol.* **2018**, *149*, 313–323, doi:10.1007/s00418-018-1640-6.
23. Ferrand, N.; Béreziat, V.; Moldes, M.; Zaoui, M.; Larsen, A.K.; Sabbah, M. WISP1/CCN4 inhibits adipocyte differentiation through repression of PPARγ activity. *Sci. Rep.* **2017**, *7*, 1–12, doi:10.1038/s41598-017-01866-2.
24. Murahovschi, V.; Pivovarova, O.; Ilkavets, I.; Dmitrieva, R.M.; Döcke, S.; Keyhani-Nejad, F.; Gögebakan, Ö.; Osterhoff, M.; Kemper, M.; Hornemann, S.; et al. WISP1 Is a novel adipokine linked to inflammation in obesity. *Diabetes* **2015**, *64*, 856–866, doi:10.2337/db14-0444.
25. Christodoulides, C.; Laudes, M.; Cawthorn, W.P.; Schinner, S.; Soos, M.; O'Rahilly, S.; Sethi, J.K.; Vidal-Puig, A. The Wnt antagonist Dickkopf-1 and its receptors are coordinately regulated during early human adipogenesis. *J. Cell Sci.* **2006**, *119*, 2613–2620, doi:10.1242/jcs.02975.
26. Gustafson, B.; Smith, U. The WNT Inhibitor Dickkopf 1 and Bone Morphogenetic Protein 4 Rescue Adipogenesis in Hypertrophic Obesity in Humans. *Diabetes* **2012**, *61*, 1217–1224, doi:10.2337/db11-1419.
27. Festy, F.; Hoareau, L.; Bes-Houtmann, S.; Péquin, A.M.; Gonthier, M.P.; Munstun, A.; Hoarau, J.J.; Césari, M.; Roche, R. Surface protein expression between human adipose tissue-derived stromal cells and mature adipocytes. *Histochem. Cell Biol.* **2005**, *124*, 113–121, doi:10.1007/s00418-005-0014-z.
28. Sidney, L.E.; Branch, M.J.; Dunphy, S.E.; Dua, H.S.; Hopkinson, A. Concise Review: Evidence for CD34 as a Common Marker for Diverse Progenitors. *Stem Cells* **2014**, *32*, 1380–1389, doi:10.1002/stem.1661.
29. Scherberich, A. A familiar stranger: CD34 expression and putative functions in SVF cells of adipose tissue. *World J. Stem Cells* **2013**, *5*, 1, doi:10.4252/wjsc.v5.i1.1.
30. Christiaens, V.; Van Hul, M.; Lijnen, H.R.; Scroyen, I. CD36 promotes adipocyte differentiation and adipogenesis. *Biochim. Biophys. Acta - Gen. Subj.* **2012**, *1820*, 949–956, doi:10.1016/j.bbagen.2012.04.001.
31. Gao, H.; Volat, F.; Sandhow, L.; Galitzky, J.; Nguyen, T.; Esteve, D.; Åström, G.; Mejhert, N.; Ledoux, S.; Thalamas, C.; et al. CD36 Is a Marker of Human Adipocyte Progenitors with Pronounced Adipogenic and Triglyceride Accumulation Potential. *Stem Cells* **2017**, *35*, 1799–1814, doi:10.1002/stem.2635.
32. Leroyer, A.S.; Blin, M.G.; Bachelier, R.; Bardin, N.; Blot-Chabaud, M.; Dignat-George, F. CD146 (Cluster of Differentiation 146): An Adhesion Molecule Involved in Vessel Homeostasis. *Arterioscler. Thromb. Vasc. Biol.* **2019**, *39*, 1026–1033, doi:10.1161/ATVBAHA.119.312653.
33. Walmsley, G.G.; Atashroo, D.A.; Maan, Z.N.; Hu, M.S.; Zielins, E.R.; Tsai, J.M.; Duscher, D.; Paik, K.; Tevlin, R.; Marecic, O.; et al. High-Throughput Screening of Surface Marker Expression on Undifferentiated and Differentiated Human Adipose-Derived Stromal Cells. *Tissue Eng. - Part A* **2015**, *21*, 2281–2291, doi:10.1089/ten.tea.2015.0039.
34. Fan, J.; Sun, Z. The Antiaging Gene Klotho Regulates Proliferation and Differentiation of Adipose-Derived Stem Cells. *Stem Cells* **2016**, *34*, 1246–1253, doi:10.1634/stemcells.2005-0235.
35. Zhang, H.; Li, Y.; Fan, Y.; Wu, J.; Zhao, B.; Guan, Y.; Chien, S.; Wang, N. Klotho is a target gene of PPAR-γ. *Kidney Int.* **2008**, *74*, 732–739, doi:10.1038/ki.2008.244.
36. Razzaque, M.S. The role of Klotho in energy metabolism. *Nat. Rev. Endocrinol.* **2012**, *8*, 579–587, doi:10.1038/nrendo.2012.75.
37. Ito, S.; Kinoshita, S.; Shiraishi, N.; Nakagawa, S.; Sekine, S.; Fujimori, T.; Nabeshima, Y.I. Molecular

cloning and expression analyses of mouse βklotho, which encodes a novel Klotho family protein. *Mech. Dev.* **2000**, *98*, 115–119, doi:10.1016/S0925-4773(00)00439-1.
38. Burl, R.B.; Ramseyer, V.D.; Rondini, E.A.; Pique-Regi, R.; Lee, Y.H.; Granneman, J.G. Deconstructing Adipogenesis Induced by β3-Adrenergic Receptor Activation with Single-Cell Expression Profiling. *Cell Metab.* **2018**, *28*, 300-309.e4, doi:10.1016/j.cmet.2018.05.025.
39. Harms, M.J.; Li, Q.; Lee, S.; Zhang, C.; Kull, B.; Hallen, S.; Thorell, A.; Alexandersson, I.; Hagberg, C.E.; Peng, X.R.; et al. Mature Human White Adipocytes Cultured under Membranes Maintain Identity, Function, and Can Transdifferentiate into Brown-like Adipocytes. *Cell Rep.* **2019**, *27*, 213-225.e5, doi:10.1016/j.celrep.2019.03.026.
40. Lee, M. Hormonal Regulation of Adipogenesis. In *Comprehensive Physiology*; John Wiley & Sons, Inc.: Hoboken, NJ, USA, 2017; Vol. 7, pp. 1151–1195.
41. Stern, J.H.; Rutkowski, J.M.; Scherer, P.E. Adiponectin, Leptin, and Fatty Acids in the Maintenance of Metabolic Homeostasis through Adipose Tissue Crosstalk. *Cell Metab.* **2016**, *23*, 770–784, doi:10.1016/j.cmet.2016.04.011.
42. Villarroya, F.; Cereijo, R.; Villarroya, J.; Giralt, M. Brown adipose tissue as a secretory organ. *Nat. Rev. Endocrinol.* **2017**, *13*, 26–35, doi:10.1038/nrendo.2016.136.
43. Caputo, T.; Tran, V.D.T.; Bararpour, N.; Winkler, C.; Aguileta, G.; Trang, K.B.; Giordano Attianese, G.M.P.; Wilson, A.; Thomas, A.; Pagni, M.; et al. Anti-adipogenic signals at the onset of obesity-related inflammation in white adipose tissue. *Cell. Mol. Life Sci.* **2020**, doi:10.1007/s00018-020-03485-z.
44. Tamucci, K.A.; Namwanje, M.; Fan, L.; Qiang, L. The dark side of browning. *Protein Cell* **2018**, *9*, 152–163, doi:10.1007/s13238-017-0434-2.
45. Wang, B.; Fu, X.; Liang, X.; Deavila, J.M.; Wang, Z.; Zhao, L.; Tian, Q.; Zhao, J.; Gomez, N.A.; Trombetta, S.C.; et al. Retinoic acid induces white adipose tissue browning by increasing adipose vascularity and inducing beige adipogenesis of PDGFRα+ adipose progenitors. *Cell Discov.* **2017**, *3*, doi:10.1038/celldisc.2017.36.
46. Nascimento, E.B.M.; Boon, M.R.; Lichtenbelt, W.D. van M. Fat Cells Gain New Identities. *Sci. Transl. Med.* **2014**, *6*, 247fs29-247fs29, doi:10.1126/scitranslmed.3009809.
47. Mortier, A.; Gouwy, M.; Damme, J. Van; Proost, P.; Struyf, S. CD26 / dipeptidylpeptidase IV — chemokine interactions : double-edged regulation of inflammation and tumor biology. **2016**, *99*, 955–969, doi:10.1189/jlb.3MR0915-401R.
48. Metzemaekers, M.; Damme, J. Van; Mortier, A.; Proost, P.; Proost, P. Regulation of Chemokine Activity – A Focus on the Role of Dipeptidyl Peptidase iv / CD26. **2016**, *7*, 1–23, doi:10.3389/fimmu.2016.00483.
49. Merrick, D.; Sakers, A.; Irgebay, Z.; Okada, C.; Calvert, C.; Morley, M.P.; Percec, I.; Seale, P. Identification of a mesenchymal progenitor cell hierarchy in adipose tissue. *Science (80-.).* **2019**, *364*, doi:10.1126/science.aav2501.
50. Rennert, R.C.; Januszyk, M.; Sorkin, M.; Rodrigues, M.; Maan, Z.N.; Duscher, D.; Whittam, A.J.; Kosaraju, R.; Chung, M.T.; Paik, K.; et al. Microfluidic single-cell transcriptional analysis rationally identifies novel surface marker profiles to enhance cell-based therapies. *Nat. Commun.* **2016**, *7*, 1–9, doi:10.1038/ncomms11945.
51. Schwalie, P.C.; Dong, H.; Zachara, M.; Russeil, J.; Alpern, D.; Akchiche, N.; Caprara, C.; Sun, W.; Schlaudraff, K.U.; Soldati, G.; et al. A stromal cell population that inhibits adipogenesis in mammalian fat depots. *Nature* **2018**, *559*, 103–108, doi:10.1038/s41586-018-0226-8.
52. Chu, A.J. Tissue Factor , Blood Coagulation, and Beyond : An Overview. **2011**, *2011*, doi:10.4061/2011/367284.
53. Brett, E.; Zielins, E.R.; Chin, M.; Januszyk, M.; Blackshear, C.P.; Findlay, M.; Momeni, A.; Gurtner, G.C.; Longaker, M.T.; Wan, D.C. Isolation of CD248-expressing stromal vascular fraction for targeted improvement of wound healing. *Wound Repair Regen.* **2017**, *25*, 414–422, doi:10.1111/wrr.12542.
54. Jossen, V.; Muoio, F.; Panella, S.; Harder, Y.; Tallone, T.; Eibl, R. An Approach towards a GMP Compliant In-Vitro Expansion of Human Adipose Stem Cells for Autologous Therapies. *Bioengineering* **2020**, *7*, 77, doi:10.3390/bioengineering7030077.
55. Sagaradze, G.; Grigorieva, O.; Nimiritsky, P.; Basalova, N.; Kalinina, N.; Akopyan, Z.; Efimenko, A.

Conditioned medium from human mesenchymal stromal cells: Towards the clinical translation. *Int. J. Mol. Sci.* 2019, *20*.

56. Lee, M.S.; Youn, C.; Kim, J.H.; Park, B.J.; Ahn, J.; Hong, S.; Kim, Y.D.; Shin, Y.K.; Park, S.G. Enhanced cell growth of adipocyte-derived mesenchymal stem cells using chemically-defined serum-free media. *Int. J. Mol. Sci.* **2017**, *18*, doi:10.3390/ijms18081779.
57. Bakopoulou, A.; Apatzidou, D.; Aggelidou, E.; Gousopoulou, E.; Leyhausen, G.; Volk, J.; Kritis, A.; Koidis, P.; Geurtsen, W. Isolation and prolonged expansion of oral mesenchymal stem cells under clinical-grade, GMP-compliant conditions differentially affects "stemness" properties. *Stem Cell Res. Ther.* **2017**, *8*, 247, doi:10.1186/s13287-017-0705-0.
58. Lensch, M.; Muise, A.; White, L.; Badowski, M.; Harris, D. Comparison of synthetic media designed for expansion of adipose-derived mesenchymal stromal cells. *Biomedicines* 2018, *6*.
59. Winnier, G.E.; Valenzuela, N.; Peters-Hall, J.; Kellner, J.; Alt, C.; Alt, E.U. Isolation of adipose tissue-derived regenerative cells from human subcutaneous tissue with or without the use of an enzymatic reagent. *PLoS One* 2019, *14*.
60. Gerby, S.; Attebi, E.; Vlaski, M.; Ivanovic, Z. A new clinical-scale serum-free xeno-free medium efficient in ex vivo amplification of mesenchymal stromal cells does not support mesenchymal stem cells. *Transfusion* **2017**, *57*, 433–439, doi:10.1111/trf.13902.
